# Integrative analysis of metabolome and transcriptome reveals regulatory mechanisms of flavonoid biosynthesis in soybean under salt stress

**DOI:** 10.3389/fpls.2024.1415867

**Published:** 2024-06-18

**Authors:** Yubin Wang, Wei Liu, Wei Li, Caijie Wang, Haiying Dai, Ran Xu, Yanwei Zhang, Lifeng Zhang

**Affiliations:** ^1^ Crop Research Institute, Shandong Academy of Agricultural Sciences, Jinan, Shandong, China; ^2^ Shandong Engineering Laboratory of Featured Crops, Jinan, Shandong, China

**Keywords:** soybean, salt stress, metabolome, transcriptome, flavonoid, regulatory mechanism

## Abstract

**Introduction:**

Salt stress is a major environmental factor that constrains soybean growth, development, and productivity. Flavonoids are key secondary metabolites that play a crucial role in enhancing plant resistance to both biotic and abiotic stress. However, a comprehensive understanding of the regulatory mechanisms underlying flavonoid biosynthesis under salt stress in soybean is lacking.

**Methods:**

In this study, an integrative analysis of soybean metabolome and transcriptome was conducted using two soybean lines, FQ03 (salt-sensitive, SS) and FQ07 (salt-tolerant, ST).

**Results:**

A total of 650 significantly changed metabolites were identified in SS and ST after salt stress treatment. Among them, 151 flavonoids were categorized into nine classes, with flavones and flavonols being the predominant flavonoid types in soybean. Heatmap analysis showed higher contents of most flavonoid metabolites in ST than in SS under salt stress, and the total flavonoid content in ST was significantly higher than that in SS. In addition, transcriptome analysis revealed a higher number of differentially expressed genes (DEGs) in ST than in SS under salt stress. KEGG enrichment analysis revealed that DEGs were mainly enriched in pathways related to phenylpropanoid biosynthesis, isoflavonoid biosynthesis, flavonoid biosynthesis, as well as flavone and flavonol biosynthesis. Notably, 55 DEGs that were mapped to the flavonoid biosynthetic pathway were identified, with most showing higher expression levels in ST than in SS. Weighted gene correlation network analysis identified eight structural genes and six transcription factor genes as key regulators of flavonoid biosynthesis within the blue module. Furthermore, qRT-PCR results confirmed the accuracy of the transcriptomic data and reliability of the identified candidate genes.

**Discussion:**

This study provides insights into the regulatory mechanisms underlying salt stress responses in soybean and highlights hub genes as potential targets for developing salt-tolerant soybean varieties.

## 1 Introduction

Soybean (*Glycine max* [L.] Merr.) is a primary source of protein and oil for both human and animal nutrition worldwide. The yield and quality of soybean is severely threatened by several abiotic stresses, including high soil salinity that is caused by poor irrigation practices, improper fertilizer application, and climate change (Yang and Guo, 2017; Long et al., 2021). According to previous studies, soybean yield decreases considerably when soil salinity level reaches 5 Ds/m (Phang et al., 2008). Moreover, salinity stress has adverse effects on soybean seedling germination and reproductive growth (Shu et al., 2017). To date, over 6% of the world’s total land area and approximately 20% of the world’s cultivated land area is affected by salinization, with the areas continuously expanding (Munns and Tester, 2008). Therefore, it is crucial to develop salinity-tolerant soybean varieties and enhance their adaptability to saline-alkali soils. Understanding the molecular mechanisms underlying salt tolerance is crucial for breeding of salt-tolerant soybean cultivars.

As a class of polyphenolic plant secondary compounds with a basic C6–C3–C6 carbon skeleton, flavonoids are widely distributed in plant flowers, fruits, leaves, roots, and other organs (Shen et al., 2022). To date, over 8000 distinct flavonoid derivatives have been discovered across different plant species and classified into various subtypes, such as flavones, flavonols, isoflavones, anthocyanins, proanthocyanidins among others, based on their structural variations (Zhuang et al., 2023). Flavonoid biosynthesis is an intricate process that involves several structural genes, such as early biosynthesis genes (EBGs) and late biosynthesis genes (LBGs) (Xu et al., 2015). The EBGs encode enzymes associated with catalyzing the production of flavonoid precursors, including phenylalanine ammonia lyase (*PAL*), cinnamate-4-hydroxylase (*C4H*), 4-coumarate:CoA ligase (*4CL*), chalcone synthase (*CHS*), and chalcone isomerase (*CHI*). The LBGs encode enzymes involved in the synthesis of anthocyanins and proanthocyanins, such as flavonoid 3’-hydroxylase (*F3’H*), flavonoid 3’5’-hydroxylase (*F3’5’H*), flavanone 3-hydroxylase (*F3H*), flavonol synthase (*FLS*), anthocyanidin synthase (*ANS*), etc. The flavonoid biosynthetic pathway and structural genes are highly conserved in plants. To date, the key genes involved in soybean flavonoid biosynthesis have been well studied and characterized. For instance, *GmIFS1*, *GmIFS2*, *GmCHS7*, and *GmCHS8* are known to play a critical role in soybean isoflavonoid biosynthesis (Jung et al., 2000; Dhaubhadel et al., 2007). Studies have shown that silencing the *GmFNSII* gene leads to a significant reduction in flavone aglycone production (Jiang et al., 2010; Yan et al., 2014). Furthermore, the overexpression of *GmCYP82D26* has been proven to lower the levels of daidzein and genistein, the two major isoflavonoid aglycones in soybean (Xia et al., 2023).

The content of flavonoids and the expression of EBGs and LBGs are not only influenced by the genetic mechanisms of plants, but also environmental stress factors, such as salt, drought, ultraviolet B radiation, temperature, and light (Bian et al., 2019; Laoué et al., 2022). High expression of *CHS*, *CHI*, and *F3H* genes has been linked to flavonoid accumulation in wheat (*Triticum aestivum* L.) under drought conditions (Ma et al., 2014). Anthocyanin content in purple kale (*Brassica oleracea* var. sabellica), as well as the expression of *C4H*, *F3H*, *DFR*, *ANS*, and *UFGT* have been shown to increase under low temperatures (Zhang et al., 2011). The roles of EBGs and LBGs in flavonoid biosynthesis and response to salt stress in tobacco (*Nicotiana tabacum* L.), *Populus euphratica*, *Arabidopsis thaliana*, have been investigated (Zhang et al., 2019a; Naing and Kim, 2021; Yu et al., 2021). Moreover, the transcriptional levels of LBGs are largely regulated by various transcription factors (TFs), such as the MBW complex consisting of MYB, bHLH transcription factors, and WD40 proteins (Liu et al., 2015). Additionally, other TFs including TTG2 (WRKY44), zinc finger proteins (TT1/WIP1), TTG16/AGL32 (MADS), and ERFs are also involved in the transcriptional regulation of LBGs (Zhou et al., 2015; Li et al., 2020).

Soybean are rich in flavonoid compounds, with isoflavones being the predominant category of flavonoids with known health benefits. The predominant isoflavones found in soybean include daidzein, genistein, and glycitein, along with their respective glucosides, acetyl-glucosides, and malonyl-glucosides (Wang and Murphy, 1994). Despite the technological advancements in metabolomics that have led to the identification of numerous flavonoid components in plants, only a few flavonoid components in soybean have been identified so far (Li et al., 2017; Zhang et al., 2019b; Aguiar et al., 2022), which hinders the study of the functional properties of flavonoid compounds in soybean. Moreover, there is limited understanding of the regulatory mechanisms underlying flavonoid biosynthesis in soybean under salt stress conditions. In this study, transcriptome and widely targeted metabolome analyses were conducted on the roots of two soybean lines, FQ03 (salt-sensitive, SS) and FQ07 (salt-tolerant, ST), under control (0 h) and salt stress conditions. The findings of this study enhance our understanding of soybean responses to salt stress and provide valuable insights into mechanisms underlying salt tolerance.

## 2 Materials and methods

### 2.1 Plant material, growth conditions, and treatments

Two soybean lines, salt-tolerant FQ07 (ST) and salt-sensitive FQ03 (SS), were selected from a group of 12 stable high-generation lines based on their salt tolerance capacities. These lines were obtained by crossing the salt-tolerant cultivar Qi Huang NO.34 (QH34) with the salt-sensitive cultivar Fan NO.13A13 (F13A13). FQ07 and FQ03 were bred and kept at the Crop Research Institute, Shandong Academy of Agricultural Sciences, Jinan, China.

Plants were cultivated in a growth chamber under the conditions of 16-h-light (26°C)/8-h-dark (22°C) photoperiod and relative humidity of approximately 55–60%. For the hydroponics experiment, soybean seeds were sterilized with 10% (v/v) sodium hypochlorite for 20 min, followed by five rinses with distilled water and then sown in sand. After seven days, the uniform seedlings were transferred to a hydroponic container filled with 5 L of half-strength Hoagland’s solution, which was refreshed every three days until the first trifoliolate leaves emerged. The seedlings were exposed to 150 mM NaCl at different time points. The roots of treated seedlings were harvested at 0 (control), 2, 6, 12 and 24 h for transcriptome sequencing, while samples collected at 0 (control), 12, and 24 h were prepared for metabolite profiling (three biological replicates per treatment). All samples were immediately frozen in liquid nitrogen and stored at −80°C until use. Shoot and root dry weight of the seedlings under control and salinity conditions were measured after six days of treatment.

For the salt tolerance assays, soybean seeds were planted in 10 cm × 10 cm round plastic pots filled with a mixture vermiculite and peat soil (ratio of 1:3, five seeds per pot, five replicates for each repeat). All pots contained an equal weight of soil. After the plants grew for 10 days, the same plants were selected for irrigate with 200 mM NaCl solution and again after six days of growth. The plant survival rate of each line was measured after 10 days of salt treatment.

### 2.2 Measurement of total flavonoid, malondialdehyde, and proline contents

Seedlings of SS and ST lines were collected after 0, 12, and 24 h of treatment with 150 mM NaCl treatment to determine the total flavonoid content, and after 0 and 72 h of treatment to determine malondialdehyde (MDA) and proline (Pro) contents. Three biological replicates were performed for each treatment, with six seedlings pooled per biological replicate. Total flavonoids content was measured using the Flavonoids Assay Kit (Solarbio, BC1330) according to the manufacturer’s instructions. The content of Malondialdehyde (MDA) was determined using the MAD Content Assay Kit (Solarbio, BC0025), and Proline (Pro) content was determined using the Proline Content Assay Kit (Solarbio, BC0290) according to the manufacturer’s instructions.

### 2.3 Determination of Na^+^ and K^+^ concentrations

The roots and leaves of the two soybean lines were collected at 0 and 72 h of treatment with 150 mM NaCl. To determine Na^+^ and K^+^ concentrations, the dried soybean root and leaf samples were ground into dry power and 0.2 g of the samples was accurately weighed into the test tubes. Thereafter, 1 M HCl was added to the samples and shaken at 28°C for 24 h. The extracts were subsequently filtered through qualitative filter papers with diameters of 9 cm. Na^+^ and K^+^ concentrations were measured using a flame photometer (FSP6620; Yuefeng Instruments & Meters Co., Ltd., Shanghai, China), according the method described by Munns et al. (2010).

### 2.4 Widely targeted metabolomic analysis

Preparation of extracts and metabolite profiling of 18 soybean root samples were carried out by Metware Biotechnology Co., Ltd. (Wuhan, China) according to the standard procedures. Briefly, each fresh root sample (0.6 g) was freeze-dried in a lyophilizer (Scientz-100F), ground into powder using a grinder, and 50 mg of the powder was dissolved in 1.2 mL 70% methanol. The mixture was vortexed once every 30 min for 30s and this was repeated six times. After centrifugation at 12,000 ×*g* for 3 min, the extracts were filtered through microporous filter membranes with pore sizes of 0.22 μm and analyzed using UPLC-ESI-MS/MS (UPLC: ExionLC™ ADsystem; MS: QTRAP 4500; both AB Sciex Pte.Ltd., Singapore) system.

Significantly changed metabolites (SCMs) were identified based on a variable importance of the projection (VIP)≥1 and a fold-change ≥2 or ≤0.5. These SCMs were subsequently annotated using the KEGG compound database (http://www.genome.jp/kegg/compound/), and then mapped to the KEGG pathway database (http://www.genome.jp/kegg/pathway.html) to determine their metabolic pathways and potential functions in soybean response to salt stress.

### 2.5 RNA sequencing and transcriptomics analysis

Total RNA was extracted from 30 soybean samples using the FastPure^®^ Universal Plant Total RNA Isolation Kit (RC411) following the manufacturer’s instructions. Subsequently, the RNA samples were sent to BGI (Shenzhen, China) for RNA sequencing (RNA-seq) analysis. Library preparation, RNA sequencing, and bioinformatics analyses were performed on an Illumina HiSeq™ 2500 platform. After removing adapters and trimming low-quality reads using SOAPnuke (version 1.6.5) (Li et al., 2008), high-quality reads were aligned to the soybean (*Glycine max*) reference genome (William 82.a4.v1) using HISAT (version 2.2.1) (Kim et al., 2015). Gene expression levels were quantified using RSEM (version 1.3.1) according to the method described by (Li et al 2011). Differentially expressed genes (DEGs) between groups (SS_0 h vs. SS_2 h; SS_0 h vs. SS_6 h; SS_0 h vs. SS_12 h; SS_0 h vs. SS_24 h; ST_0 h vs. ST_2 h; ST_0 h vs. ST_6 h; ST_0 h vs. ST_12 h; ST_0 h vs. ST_24 h) were identified based on |log2(fold-change)| ≥ 2 and q-value (FDR, padj) ≤ 0.05 using DESeq2 (Wang et al., 2009). Gene Ontology (GO) and KEGG enrichment analysis were performed using phyper in R software and the TermFinder package, with a Q value threshold of ≤ 0.05.

### 2.6 Weighted gene co-expression network analysis

Co-expression network analysis was constructed using the WGCNA R package (v 3.5.1) developed by Langfelder and Horvath in 2008. A total of 7963 DEGs were used as the input file, with 20 types of flavonoids serving as the trait file for generating the co-expression network and modules. Network construction and module identification were performed based on the topological overlap metrix similarity. The resulting modules were then used to evaluate the relationships between gene expression levels and flavonoid abundances across the 20 samples. The co-expression network map of flavonois-associated genes and metabolites was visualized using Cytoscape software (version v3.9.1).

### 2.7 Principal component analysis and canonical correlation analysis

Principal component analysis (PCA) and canonical correlation analysis (CCA) analyses were conducted using the Metware Cloud Platform (https://cloud.metware.cn/). CCA was performed to determine potential correlations between flavonoid synthesis genes and metabolites. The contents of 20 flavonoids and expression levels of 55 DEGs associated with the flavonoid biosynthetic pathway in the blue module were used as inputs for CCA with default parameters.

### 2.8 Quantitative reverse transcription polymerase chain reaction

Eight genes (*GmCYP81Es*, *GmFLS*, *Gm4CL*, *GmMYBs*, and *GmbHLHs*) were selected for the verification of the transcriptome results by quantitative reverse transcription polymerase chain reaction (qRT-PCR). Three technical replicates were used for each sample. The qRT-PCR analysis was performed on a Light Cycler 480 (Roche, Mannheim, Germany) platform using Takara SYBR Premix Extaq (Takara, Bio., Inc., Kusatsu, Japan). The reaction conditions and reaction system were set according to the manufacturer’s instructions. The relative gene expression levels in different soybean samples were calculated using the 2^−ΔΔ^Ct method (Livak and Schmittgen, 2001). *GmActin* (Glyma.18G290800) was used as an internal reference gene (Liu et al., 2024), and the specific primers are listed in **Supplementary Table 1**.

### 2.9 Statistical analysis

Data were analyzed using SAS 8.0 (SAS Institute Inc., Cary, NC, USA). The student’s t test was used for the comparisons between two groups of date.

## 3 Results

### 3.1 Phenotypic analysis of the FQ03 (SS) and FQ07 (ST) in response to salinity stress

**Figure 1:**
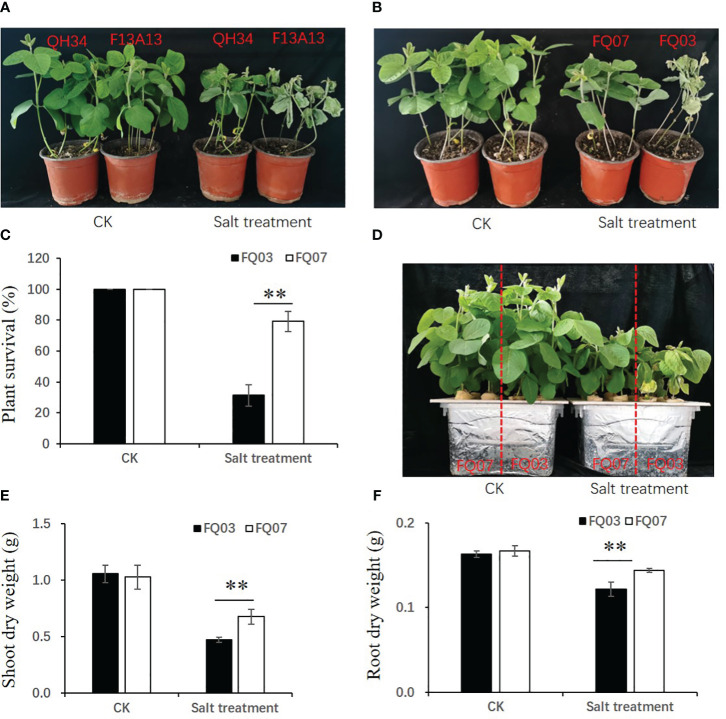
Morphological changes of soybean lines FQ07 (salt-tolerant, ST) and FQ03 (salt-sensitive, SS) in response to salinity stress. **(A)** Phenotypic differences between two soybean cultivars QH34 and F13A13 under control and 200 mM NaCl treatments. **(B)** Phenotypic differences between two soybean lines FQ03 and FQ07 under control and 200 mM NaCl treatments. **(C)** Plant survival rate of FQ03 and FQ07 under control and 200 mM NaCl treatments. **(D)** Hydroponic phenotype of FQ03 and FQ07 under control and 150 mM NaCl treatments. **(E, F)** Shoot dry weight **(E)** and root dry weight **(F)** of FQ03 and FQ07 under control and 150 mM NaCl treatments. Values with error bars represent mean ± SD (n=6). Statistical significance was determined using Student’s t test. **, P ≤ 0.01.

The salt sensitivity of two soybean varieties, QH34 and F13A13, was evaluated under control and salt stress conditions. The phenotypes are depicted in **Figure 1A**, after 10 days of salt treatment, the leaves of QH34 remained green, but most leaves of F13A13 turned yellow or died. This indicates that QH34 exhibited stronger resistance to salinity stress compared to F13A13. Twelve high generation lines were obtained by crossing QH34 and F13A13, and their salt tolerance capacities were evaluated (**Supplementary Figure 1**). The results showed that FQ07 exhibited high salt tolerance and survival rate, whereas FQ03 was salt-sensitive and had a low survival rate. Specifically, 87.5% of FQ07 and 16.67% of FQ03 plants survived under salt stress (**Figures 1B, C**). To further confirm the salt sensitivity of FQ03 and FQ07, seedlings were cultivated in Hoagland’s solution with and without NaCl, respectively. The growth of FQ03 and FQ07 was inhibited after six days of salt treatment, but FQ07 exhibited a superior salt tolerance capacity than FQ03. FQ03 leaves turned yellow, while FQ07 leaves remained green (**Figure 1D**). Additionally, shoot and root dry weights of FQ07 seedlings were significantly higher than those of FQ03 seedlings after salt stress treatment (**Figures 1E, F**). Therefore, FQ03 was identified as a salt-sensitive (SS) genotype, whereas FQ07 as a salt-tolerant (ST) genotype for further comparative transcriptomic and metabolomic analyses.

**Figure 2:**
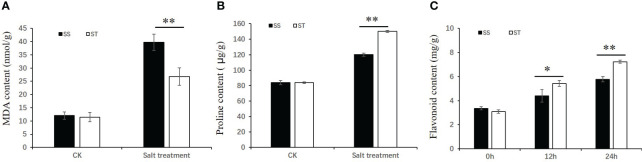
Physiological performance of soybean lines SS (Salt-sensitive) and ST (Salt-tolerant) in response to salinity stress. **(A, B)** The malondialdehyde **(A)** and proline **(B)** contents of the SS and ST grown under control and 150 mM NaCl treatment for 72 h. **(C)** The flavonoid content of the SS and ST grown under control and 150 mM NaCl treatment for 12 h and 24 h. Values with error bars represent mean ± SD (n=3). Statistical significance was determined using Student’s t test. *, P ≤ 0.05, **, P ≤ 0.01.

To investigate the impact of salt stress on soybean, levels of malondialdehyde (MDA), proline, and total flavonoid content were assessed (**Figure 2**). The results indicated that MDA content in SS and ST under control conditions was similar, but considerably higher in SS than in ST under salt stress (**Figure 2A**). Conversely, proline content in ST was significantly higher than that in SS after exposure to salt stress (**Figure 2B**). Na^+^ concentrations in the shoots and roots of ST were significantly lower than those of SS after salt treatment, while K^+^ concentrations in the roots of ST were significantly higher than those of SS under both control and salt stress treatment (**Supplementary Figure 2**). The total flavonoid content was also analyzed. The results showed that the total flavonoid content significantly increased after salt treatment, rising from 3.36 mg/g to 4.39 mg/g and 5.77mg/g after 12 h and 24 h salt treatment in SS, respectively. In FQ07, the total flavonoid content increased from 3.09 mg/g to 5.42 mg/g and 7.22 mg/g after 12 h and 24 h of salt treatment, respectively (**Figure 2C**).

### 3.2 Metabolome analysis of SS and ST under salt stress

**Figure 3:**
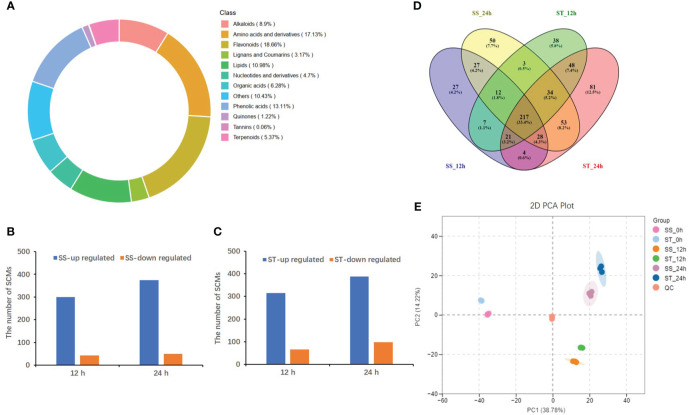
Analysis of significantly changed metabolites (SCMs) under salt stress in SS and ST. **(A)** Classifications and proportions of 650 SCMs detected in SS and ST. **(B, C)** The number of SCMs in SS **(B)** and in ST **(C)** after 12 h and 24 h 150 M NaCl treatment. **(D)** Venn diagram showing SCMs identified between SS and ST after 12 h and 24 h 150 M NaCl treatment. **(E)** PCA plot of 18 samples based on the metabolites abundances.

A total of 650 SCMs were identified in both SS and ST plants based on a variable importance of the projection (VIP)≥1 and a fold-change ≥2 or ≤0.5 (**Supplementary Table 2**). These SCMs were categorized into 12 groups, with flavonoids (18.66%), amino acids and derivatives (17.13%), and phenolic acids (13.11%) accounting for the highest proportion of the metabolites (**Figure 3A**). Furthermore, 343/380 (300/314 up accumulated and 43/66 down accumulated) and 424/486 (374/388 up accumulated and 50/98 down accumulated) SCMs were identified in SS and ST at 12 and 24 h, respectively, when compared to the control (0 h) (**Figures 3B, C**). Additionally, a total of 284 and 320 common metabolites accumulated in SS and ST, respectively. Among these, 103 metabolites were specific to ST, while 217 metabolites overlapping between SS and ST (**Figure 3D**; **Supplementary Figure 3**). Principal component analysis (PCA) of the 18 samples, including three replicates distinctly separated the samples into the first two principal components, which accounted for 38.78% (PC1) and 14.22% (PC2) of the variance, respectively (**Figure 3E**). A heatmap of all SCMs revealed significant differences in metabolite levels between SS and ST under salt stress (**Supplementary Figure 4**). Orthogonal partial least squares-discriminant analysis (OPLS-DA) was conducted to determine the differences between SS_0 h vs. SS_12 h (Q2 = 0.981), SS_0 h vs. SS_24 h (Q2 = 0.987), ST_0 h vs. ST_12 h (Q2 = 0.979), as well as ST_0 h vs. ST_24 h (Q2 = 0.986) (**Supplementary Figure 5**). The high Q2 values (>0.9) suggested that the OPLS-DA models were stable and reliable.

**Figure 4:**
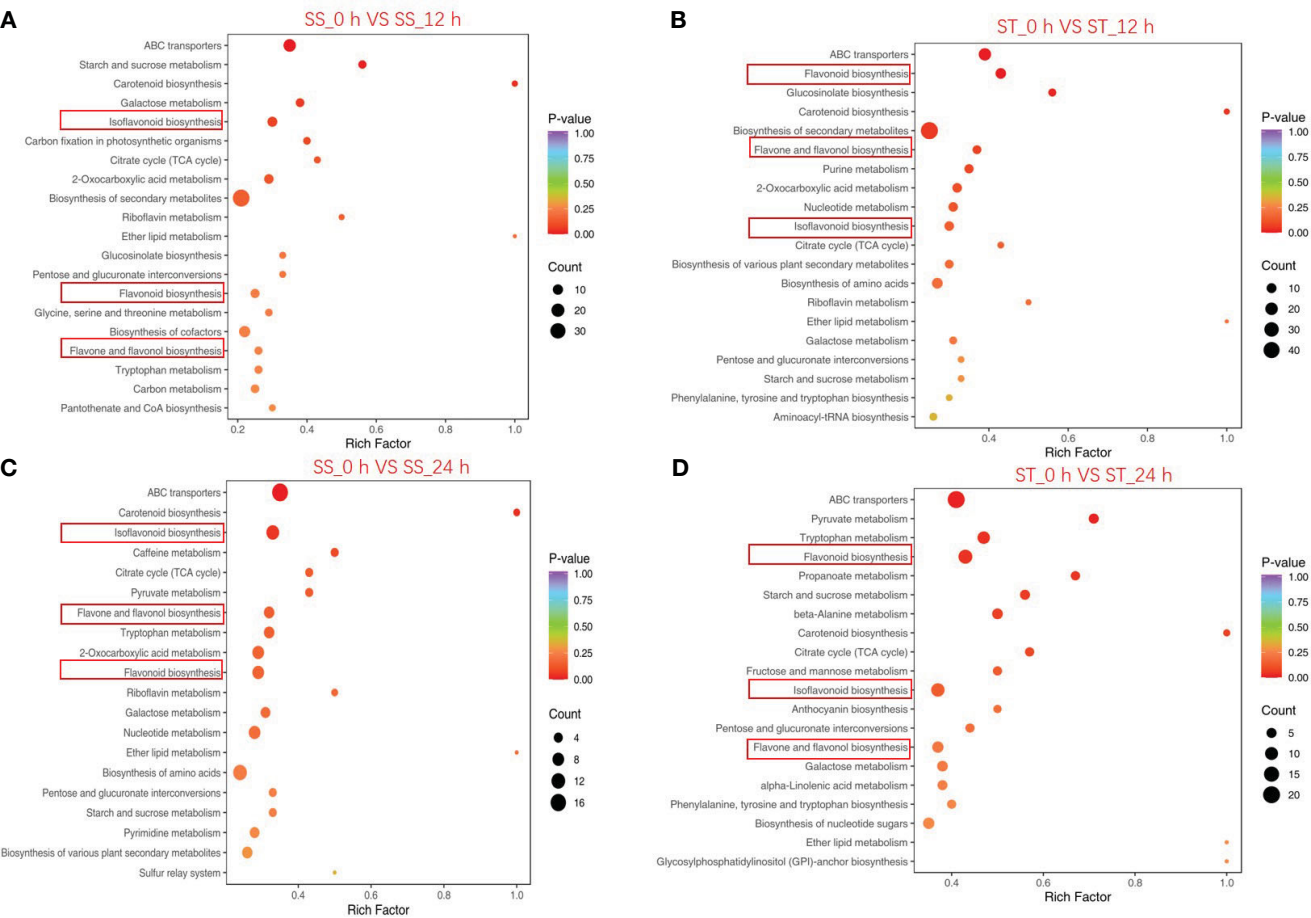
KEGG enrichment analysis of significantly changed metabolites (SCMs) between the comparison groups. SS_0 h vs. SS_12 h **(A)**, ST_0 h vs. ST_12 h **(B)**, SS_0 h vs. SS_24 h **(C)**, and ST_0 h vs. ST_24 h **(D)**. The abscissa in the figure is the ratio of the number of SCMs annotated on the KEGG pathway to the total number of SCMs, and the ordinate is the KEGG pathway name. The depth of color represents the degree of enrichment.

The top 20 significantly enriched metabolic pathways identified by KEGG pathway enrichment analysis are illustrated in **Figure 4**. The results revealed that flavonoid biosynthesis, isoflavonoid biosynthesis, and flavone and flavonol biosynthesis were significantly enriched in all of the four comparisons (SS_0 h vs. SS_12 h; SS_0 h vs. SS_24 h; ST_0 h vs. ST_12 h; ST_0 h vs. ST_24 h). The results suggest that the differentially accumulated metabolites associated with these pathways contributed to the variations in flavonoid contents between SS and ST after salt stress treatment.

### 3.3 Flavonoids in SS and ST under salt stress

**Figure 5:**
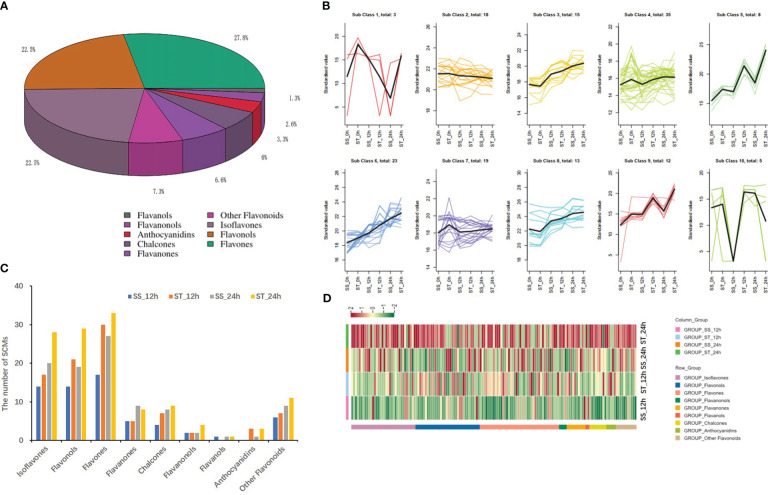
Analysis of differentially flavonoid metabolites under salt stress in SS and ST. **(A)** Classifications and proportions of 151 flavonoids detected in soybean. **(B)** Trend analysis of differentially flavonoid metabolites in SS and ST. **(C)** Comparison the number of flavonoids in different samples. **(D)** Heat map of flavonoid metabolites by Tbtools.

A total of 151 flavonoid compounds were identified and classified into nine classes based on modifications to the C6–C3–C6 carbon skeleton. These classes included 42 flavones, 34 isoflavones, 34 flavonols, 10 flavanones, 9 chalcones, 5 anthocyanidins, 4 flavanonols, 2 flavanols, and 11 other flavonoids, with flavones, flavonols, and isoflavones being the most abundant (**Figure 5A**; **Supplementary Table 3**). Among them, 8-isopentenyl daidzein (Lagp007098) was the most abundant flavonoid in soybean, followed by Apigenin-7-*O*-glucoside (MWSHY0189), and Licoflavone A (MWSHY0164). The K-means analysis divided all flavonoid compounds into 10 clusters, with seven clusters (class 3, class 4, class 5, class 6, class 8, and class 9) showing a consistent variation trend with the total flavonoid content (**Figure 5B**). A comparison of flavonoids in the two soybean lines revealed that there were more flavonoids in ST than in SS, particularly after 24 h of salt stress treatment. Specifically, 63, 92, 96, and 126 compounds showed differential accumulation between SS_0h vs. SS_12h, ST_0h vs. ST_12h, SS_0h vs. SS_24h, and ST_0h vs. ST_24h (**Figure 5C**), which possibly led to the variations in total flavonoid content. A total of 52 SCMs in the four comparisons overlapped, while 38 and 44 SCMs were specifically identified in ST after 12 and 24 h of salt treatment, respectively (**Supplementary Figure 6**). Furthermore, the number of flavonoids in ST_12 h/24 h was higher than that in SS_12 h/24 h, indicating the potential role of flavonoids in salt stress response in soybean. Volcano plots showed that the number of up accumulated flavonoids in both ST and SS was higher than that of down accumulated flavonoids after 12 and 24 h of salt stress treatment (**Supplementary Figure 7**). Heatmap analysis revealed that the contents of most flavonoids in ST increased significantly when compared to those in SS, particularly after 24 h of salt stress treatment (**Figure 5D**).

### 3.4 Transcriptomic analysis of SS and ST under salt stress

After quality control, the libraries generated an average of approximately 6.64 Gb of clean bases and 44.28 million clean reads per sample. The Q20 and Q30 values for all libraries exceeded 95.67% and 89.21%, respectively. The average mapping ratio with the reference genome was 86.55% (**Supplementary Table 4**).

**Figure 6:**
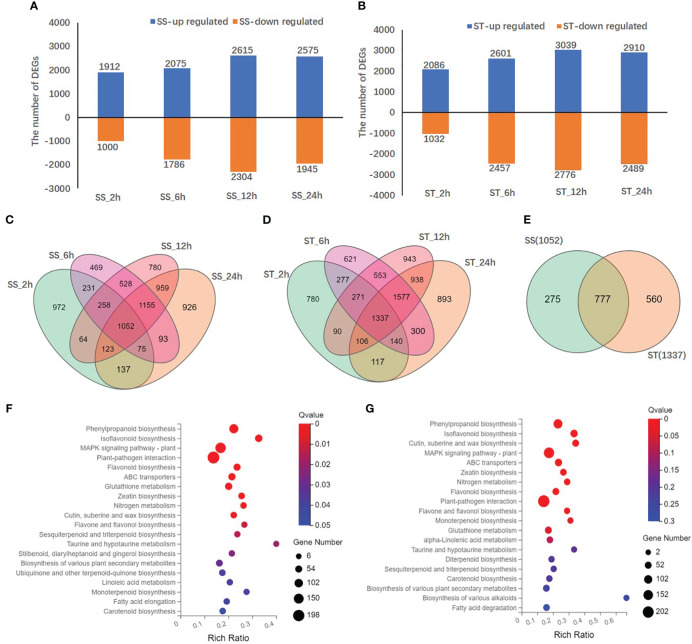
Differentially expressed genes (DEGs) in SS and ST in response to salt stress. **(A, B)** The numbers of DEGs in SS **(A)** and ST **(B)** at different salt stress time points. **(C, D)** Venn diagrams of DEGs among different salt stress time points in SS **(C)**, ST **(D)**, at 2, 6, 12 and 24 h after salt treatment. **(E)** Venn diagrams of common DEGs in SS and ST at four time points. **(F, G)** KEGG enrichment analysis of DEGs between the comparison groups **(F)** SS_0 h vs. SS_24 h, **(G)** ST_0 h vs. ST_24 h. Each bubble in the plot represents a metabolic pathway. The abscissa in the figure represents the ratio of the number of DEGs annotated on the KEGG pathway to the total number of DEGs, and the ordinate represents the KEGG pathway name. The color depth indicates the degree of enrichment.

A total of 10224 DEGs were identified at four different time points (**Supplementary Table 5**). Compared to the control (0 h), a total of 2912/3118, 3861/5058, 4919/5815, and 4520/5399 DEGs were identified in SS/ST at 2, 6, 12, and 24 h, respectively (**Figures 6A, B**). Specifically, 2086, 2601, 3039, and 2910 DEGs were upregulated in ST, whereas 1032, 2457, 2776, and 2489 DEGs were downregulated in ST under salt stress and control at 2, 6, 12, and 24 h, respectively (**Figure 6A**). However, 1912, 2075, 2615, and 2575 DEGs were upregulated, and 1000, 1786, 2304, and 1945 DEGs were downregulated in SS at the same time points (**Figure 6B**). The results revealed that the number of DEGs in ST was higher than that in SS at all time points, with the number of upregulated DEGs being higher than that of downregulated DEGs. In addition, a total of 1052 and 1337 common DEGs were identified in SS and ST, respectively, at 2, 6, 12, and 24 h (**Figures 6C, D**). Among them, 560 DEGs were specific to ST and 777 DEGs overlapped between SS and ST (**Figure 6E**). In particular, 1038, 1894, 1623, and 1781 DEGs were exclusively expressed in ST and 2080, 3182, and 4192 DEGs in SS, while 2080, 3182, 4192, and 3627 DEGs overlapped between SS and ST at 2, 6, 12, and 24 h, respectively (**Supplementary Figure 8**).

According to gene ontology (GO) annotations, a total of 7912 DEGs were categorized into biological process, cellular component, and molecular function (**Supplementary Figure S9**). Most DEGs in the biological process category were annotated under cellular process (2495) and metabolic process (2151). The most common GO terms within the cellular component category were membrane (2899) and membrane part (2720). For the molecular function category, most DEGs were annotated under catalytic activity (3793) and binding (3727). Kyoto encyclopedia of genes and genomes (KEGG) enrichment analysis revealed the top 20 enriched metabolic pathways, including phenylpropanoid biosynthesis (ko00940), MAPK signaling pathway (ko4016), isoflavonoid biosynthesis (ko00943), flavonoid biosynthesis (ko00941), and flavone and flavonol biosynthesis (ko00944), as presented in a bubble diagram (**Figures 6F, G**; **Supplementary Figure S10**). The results indicate that the transcription levels of flavonoid biosynthesis genes in SS and ST were influenced by salt stress.

### 3.5 Co-expression network analysis associated with flavonoid biosynthesis under salt stress

**Figure 7:**
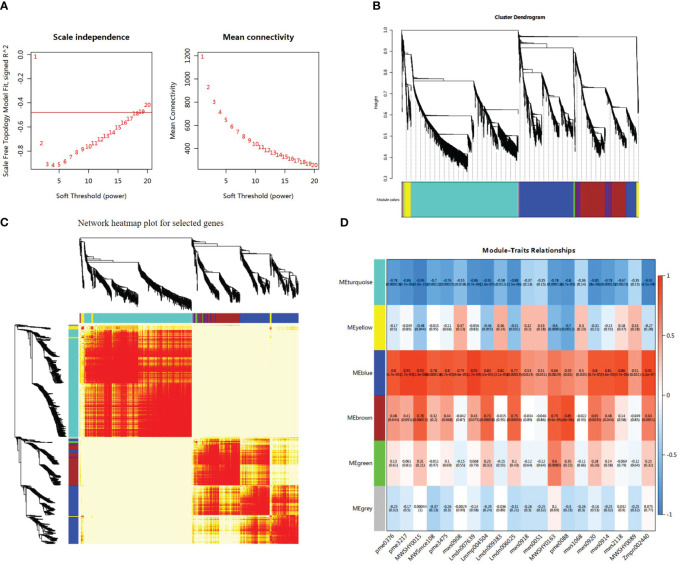
Integrative analysis of significantly changed metabolites (SCMs) and differentially expressed genes (DEGs) under salt stress in SS and ST. **(A)** Determination of soft threshold. The abscissa represents the soft threshold (β). Ordinate corresponds to the index of scale free network model. The average link degree of each soft threshold. **(B)** Network heatmap plot of genes subjected to co-expression module calculation. **(C)** Module heatmap. **(D)** Module-trait relationship heat map. Red indicates high correlation, and blue indicates low correlation.

A heatmap illustrating the module–trait relationship was generated using 10224 DEGs and 20 types of flavonoids. A soft threshold (power) of 20 was applied to construct the gene co-expression network (**Figure 7A**). The DEGs were categorized into six co-expression modules that were distinguished by distinct colors based on their expression profiles (**Figures 7B, C**). According to correlation analysis of the ‘module character’, the blue module displayed a significant positive correlation with 14 flavonols and exhibited the highest correlation with four specific flavonoids: isoliquiritigenin, liquiritigenin, 3,9-dihydroxypterocarpan, and 3,4,2’,4’,6’-pentahydroxychalcone (r > 0.9, p < 0.001) (**Figure 7D**). There were 2599 DEGs and 264 TFs, along with 28 flavonoid synthesis genes in this module. In addition, the brown module exhibited a positive correlation with luteolin content (r = 0.85, p < 0.001) and dihydrokaempferol content (r = 0.79, p < 0.001). The DEGs in the blue module were selected for further analysis. Heatmap analysis based on fragments per kilobase million (FPKM) values indicated that the expression levels of most genes in the blue module increased under salt stress, with ST exhibiting higher expression levels than SS (**Supplementary Figure 11**). This expression pattern was consistent with the variations in total flavonoid content. GO analysis of DEGs in the blue module revealed that the DEGs were significantly enriched in cellular and metabolic process under the biological process category, as well as binding and catalytic activities under the molecular function category (**Supplementary Figure 12A**). KEGG enrichment analysis revealed that DEGs in the blue module were significantly enriched in isoflavonoid biosynthesis, phenylpropanoid biosynthesis, and flavonoid biosynthesis pathways (**Supplementary Figure 12B**).

**Figure 8:**
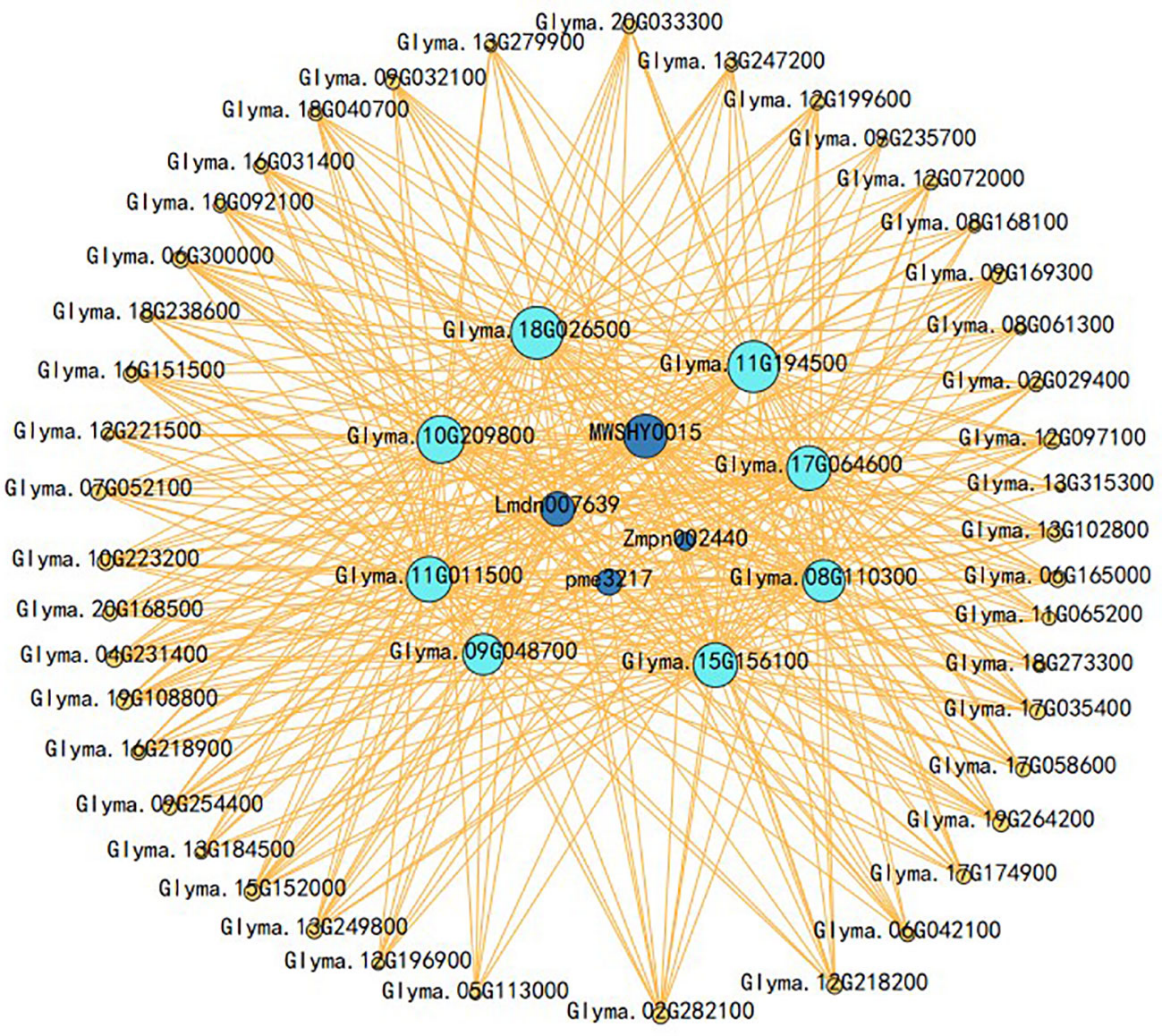
Co-expression network analysis between flavonoid contents and differentially expressed genes (DEGs) related to flavonoid metabolism under salt stress in the blue module. Purple circles represent TFs, light blue circles represent structural genes, and dark blue circles represent flavonoids.

The gene co-expression network was constructed based on the strong positive correlations of the genes with four classes of flavonoids in the blue module (**Figure 8**). The outer layer of the gene co-expression network diagram consisted of 43 TF genes (12 *MYBs*, 8 *NACs*, 6 *bHLHs*, 5 *WRKYs*, 4 *ERFs*, 3 *HD-ZIPs*, 1 *C2H2*, 1 *bZIP*, 1 *HSF*, 1 *LBD*, and 1 *GRAS*) that were identified based on their strong correlations (r > 0.95) with the four flavonoids (**Supplementary Tables 6**, **7**). Among these, the expression levels of six TFs [three *MYBs* (Glyma.19G264200, Glyma.09G032100, and Glyma.18G273300), one *bHLH* (Glyma.08G061300), one *ERF* (Glyma.20G168500), and one *NAC* (*Glyma.09G235700*)] in ST were significantly higher than those in SS. Similarly, eight flavonoid biosynthesis genes were identified in the middle of the co-expression network diagram, including two *GmCYP81Es* (Glyma.09G048700 and Glyma.15G156100), two *GmCHSs* (Glyma.08G110300 and Glyma.11G011500), one *GmFLS* (Glyma.18G026500), one *GmPAL* (Glyma.10G209800), and two *Gm4CLs* (Glyma.11G194500 and Glyma.17G064600).

### 3.6 Gene expression and metabolite accumulation in the flavonoid biosynthetic pathway

**Figure 9:**
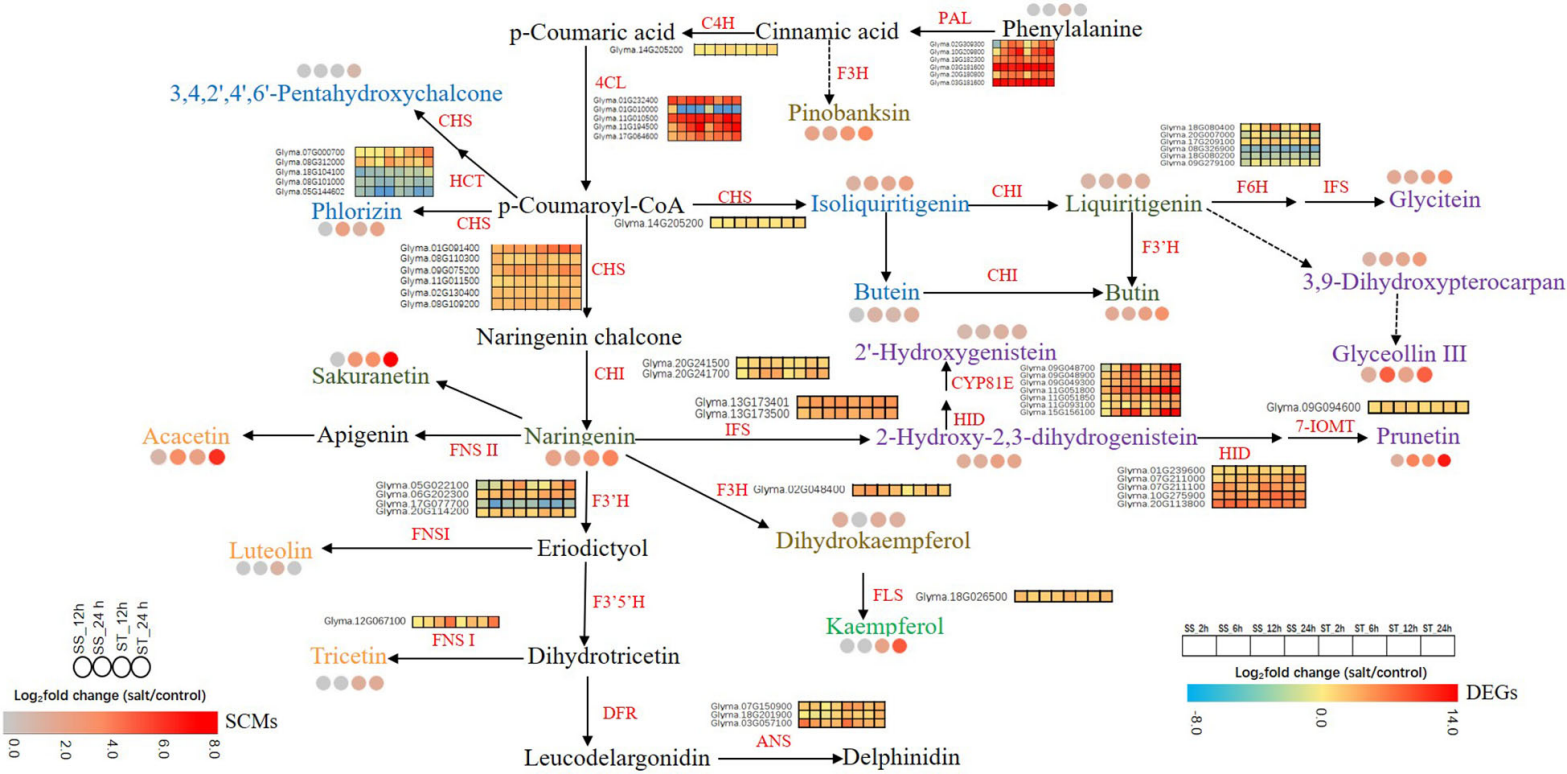
Flavonoid biosynthesis pathway in soybean from SS and ST under salt stress. Gene expression and flavonoid content are shown in heatmaps based on the log_2_ (fold change) of the DEGs and SCMs. For the gene expression, red indicates low expression, and blue indicates high expression. For the flavonoid content, high abundance are indicated in red, and those with low abundance are indicated in white. PAL, phenylalanine ammonia lyase; C4H, cinnamate 4-hydroxylase; 4CL, 4-coumaroyl CoA ligase; CHS, chalcone synthase; CHI, chalcone isomerase; F3’H, flavonoid 3’-hydroxylase; F3’5’H, flavonoid 3’,5’-hydroxylase; ANS, anthocyanidin synthase; HCT, hydroxycinnamoyltransferase; HID, 2-hydroxyisoflavanone dehydratase; 7-IOMT, naringenin-7-O-methyltransferase; CYP81E, isoflavone 2’-and 3’-hydroxylases; IFS, 2-hydroxyisoflavanone synthase; DFR, dihydroflavonol reducatse; F6H, flavonoid 6-hydroxylase; FNS, flavone synthase; FLS, flavonol synthase; F3H, flavanone 3 b-hydroxylase.

A pathway diagram illustrating the DEGs and SCMs in the two soybean lines was generated based on the flavonoid biosynthetic pathway described in model plants. A total of 20 types of flavonoids and 55 structural genes were mapped to the flavonoid biosynthetic pathway. Specifically, 11 structural genes (five *GmPALs*, one *GmC4H*, and five *Gm4CLs*) were rassociated with the phenylpropanoid pathway, while 44 structural genes (six *GmCHSs*, two *GmCHIs*, three *GmANSs*, three *GmFNSIs*, three *GmFLS*, five *GmHCTs*, three *GmIFSs*, one *Gm7-IOMT*, seven *GmCYP81Es*, five *GmHIDs*, six *GmF6Hs*, four *GmF3’H*, and one *GmFLS*) were associated with the flavonoid biosynthetic pathway (**Supplementary Table 8**). Metabolomic analysis showed that the accumulation levels of six isoflavones (prunetin, 2-hydroxy-2,3-dihydrogenistein, 2’-hydroxygenistein, glyceollin III, 3,9-dihydroxypterocarpan, and glycitein), four flavanones (sakuranetin, naringenin, butin, and liquiritigenin), four chalcones (isoliquiritigenin, butein, 3,4,2’,4’,6’-pentahydroxychalcone, and phlorizin), three flavones (luteolin, tricetin, and acacetin), two flavanonols (pinobanksin and dihydrokaempferol), and one flavonol (kaempferol) increased significantly in one or both genotypes under salt stress, implying that the accumulation of flavonoids is crucial in soybean response to salt stress (**Figure 9**; **Supplementary Table 9**). In particular, most flavonoids in ST, including kaempferol, naringenin, butin, 2-hydroxy-2,3-dihydrogenistein, prunetin, acacetin, sakuranetin, and pinobanksin exhibited significantly higher levels than those in SS. Additionally, the levels of four flavonoids in ST (kaempferol, luteolin, tricetin, and 3,4,2’,4’,6’-pentahydroxychalcone) increased substantially after 12 or 24 h of salt stress treatment (**Figure 9**; **Supplementary Table 9**). Notably, tricetin content in ST increased by 1.6- and 1.7-fold after 12 and 24 h of salt stress treatment, respectively, whereas kaempferol content in ST increased by 2.3- and 5-fold after 12 and 24 h of salt stress treatment, respectively.

The expression levels of genes associated with the phenylpropanoid and flavonoid biosynthetic pathways are shown in **Figure 9** and **Supplementary Table 8**. Most of the genes associated with flavonoid biosynthesis were upregulated in both SS and ST under salt stress. Naringenin, a core metabolite in the flavonoid biosynthetic pathway, can be converted into various compounds such as 2-hydroxy-2,3-dihydrogenistein by IFS, kaempferol by F3H and FLS, 2’-hydroxygenistein by HID and CYP81E, and prubetin by HID and 7-IOMT. The higher expression levels of *GmFLS* (Glyma.18G026500), *GmIFSs* (Glyma.13G173500), *GmHIDs* (Glyma.07G211000 and Glyma.10G275900), and *GmCYP81Es* (Glyma.09G048700, Glyma.11G051800, Glyma.11G051850, Glyma.11G093100, and Glyma.15G156100) in ST than in SS after 12 or 24 h of exposure to salt stress could explain the increased levels of kaempferol, 2-hydroxy-2,3-dihydrogenistein, prunetin, and 2’-hydroxygenistein. p-Coumaroyl-CoA is synthesized from cinnamoyl-CoA, it can be transformed into phlorizin and isoliquiritigenin by CHS, and 3,4,2’,4’,6’-pentahydroxychalcone by HCT and CHS. The higher expression levels of three *GmCHSs* (Glyma.01G091400, Glyma.08G110300, and Glyma.11G011500) and two *GmHCTs* (Glyma.07G000700 and Glyma.08G312000) in ST than in SS could explain the high contents of isoliquiritigenin, 3,4,2’,4’,6’-pentahydroxychalcone, and phlorizin in ST.

### 3.7 Correlation and canonical correlation analyses of the expression levels of flavonoid synthesis genes and flavonoid contents

**Figure 10:**
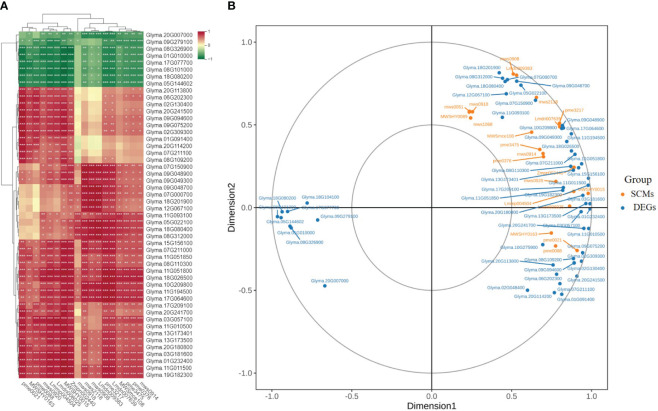
Screening of the main contributing genes for flavonoid biosynthesis in SS and ST. **(A)** Intergroup correlation analysis of 20 flavonoids and 55 flavonoid synthesis-related genes. **(B)** Canonical correlation analysis (CCA) of 20 flavonoids and related synthetic genes between SS and ST in soybean.

Correlation analysis was conducted to determine the relationships between the expression levels of 55 structural genes and the contents of 20 types of flavonoids. The results indicated that liquiritigenin and 3,4,2’,4’,6’-pentahydroxychalcone exhibited positive correlations with all structural genes. In addition, 18 flavonoids showed notable correlations with specific genes, such as *GmCYP81Es* (Glyma.11G051800 and Glyma.15G56100), *GmFLS* (Glyma.18G026500), *GmPAL* (Glyma.10G209800), and two *Gm4CLs* (Glyma.11G194500 and Glyma.17G064600) (**Figure 10A**). The CCA results showed significant correlations between the expression levels of *GmCYP81Es* (Glyma.11G051800 and Glyma.11G051850) and *GmHID* (Glyma.07G211000) with the content of 2’-Hydroxygenistein. The expression level of *GmIFS* (Glyma.13G173500) was significantly correlated with 2-hydroxy-2,3-dihydrogenistein content. Additionally, the expression levels of *GmANS* (Glyma.18G201900) and *GmHCT* (Glyma.07G000700) were significantly associated with the content of glyceollin III. Lastly, the expression levels of *GmPAL* (Glyma.10G209800), *GmCYP81E* (Glyma.09G048900), and *Gm4CL* (Glyma.17G064600) were significantly linked to the contents of isoliquiritigenin and 3,9-dihydroxypterocarpan (**Figure 10B**).

### 3.8 Verification of DEGs involved in flavonoid synthesis using qRT-PCR

Eight DEGs, including four genes involved in flavonoid biosynthesis (two *CPY81Es*, one *4CL*, and one *FLS*) and four TF genes (two *MYBs* and two *bHLHs*) identified from the WGCNA co-expression network were analyzed by qRT-PCR. The relative expression levels of the selected DEGs were consistent with those of RNA-Seq data (**Supplementary Figure 13**), thereby confirming the accuracy and repeatability of the RNA-Seq data.

## 4 Discussion

A common feature associated with abiotic stress is the accumulation of reactive oxygen species (ROS). As stress-responsive metabolites, flavonoids play a crucial role in alleviating oxidative damage caused by abiotic stress by reducing various forms of ROS (Dong and Lin, 2021; Salika and Riffat, 2021). Previous studies have demonstrated that salt stress enhances the accumulation of flavonoids in *Apocynum venetum* (Xu et al., 2020), *Oryza sativa* (Gazara et al., 2020), and *Sorghum bicolor* (Ren et al., 2022). In this study, we observed a significant increase in total flavonoid content in both SS and ST lines after salt stress treatment (**Figure 2C**), indicating that salt stress induced ROS accumulation, in turn, increasing flavonoid content, which protects plant cells from oxidative damage. Notably, the total flavonoid content in ST plants was higher than that in SS plants after salt stress treatment, suggesting that salt-tolerant plants have a robust ROS-scavenging capacity, which mitigates ROS-induced cell damage under salt stress conditions. Moreover, MDA content in ST was significantly lower than that in SS under salt stress treatment (**Figure 2A**). However, Pro content in ST was higher than that in SS under salt stress treatment (**Figure 2B**). These findings demonstrate that ST plants accumulate more flavonoids under salt stress, thereby enhancing their ability to reduce cell damage caused by salt stress and resistance to salt stress.

Although previous studies on flavonoids in soybean have been conducted, the number of flavonoid components identified remains low (Zhang et al., 2019b; Aguiar et al., 2022). Our study identified 151 flavonoid components in soybean roots, representing a significant supplement to the previous work. Flavonoids are largely recognized as primary antioxidants in plants. Luteolin, a major flavone aglycone and natural antioxidant, can effectively scavenge free radicals and protect plant cells (Seelinger et al., 2008). Luteolin accumulation has been shown to enhance salt stress tolerance in *Beta vulgaris* (sugar beet) (Liu et al., 2020). Similarly, kaempferol has been reported to have the potential to reduce oxidative damage by scavenging ROS generated under drought, salt, and heat stress (Jan et al., 2022). In addition, butin (7,3’,4’-trihydroxyflavanone), which is a product of daidzein metabolism, is a representative isoflavone in soybean with anti-inflammatory activity (Kim et al., 2020). In this study, luteolin, kaempferol, and butin contents in ST were higher than those in SS, indicating that ST accumulated more flavonoids under salt stress, thereby enhancing its antioxidant capacity. Isoflavonoids are predominantly synthesized in soybean and other leguminous plants, where they function as antioxidants and improve plant responses to biotic and abiotic stresses (Sepiol et al., 2017). According to our results, the contents of three isoflavonoids (prunetin, 2-hydroxy-2,3-dihydrogenistein, and 3,9-dihydroxypterocarpan) in ST were significantly higher than those in SS, suggesting their crucial role in enhancing soybean responses to salt stress. Furthermore, higher contents of prunetin, naringenin, 3,9-dihydroxypterocarpan were observed in ST than in SS. These flavonoids and their roles in abiotic stress have not been previously reported, which requires further studies. Certain flavonoids, such as 3,4,2’,4’,6’-pentahydroxychalcone and 2-hydroxy-2,3-dihydrogenistein, were identified for the first time in soybean. Therefore, our study enriches and supplements previous work on the flavonoid biosynthetic pathway in soybean.

Transcriptomic data can provide valuable insights into key genes associated with specific metabolic pathways. In this study, KEGG enrichment analysis revealed significant changes in the expression of genes associated with the flavonoid biosynthetic pathway in SS and ST under salt stress (**Figure 4**). WGCNA revealed a blue module that exhibited a strong positive correlation with 14 flavonols (**Figure 7D**). A total of 43 TFs and 8 structural genes were strongly correlated with flavonoid biosynthesis (**Figure 8**). Flavonol synthase (FLS) is a key enzyme in flavonol biosynthesis and it catalyzes the conversion of dihydrokaempferol to kaempferol (Tian et al., 2015). Previous studies have shown that activation of *FLS*s expression increases kaempferol content in *Gerbera hybrida* (Zhong et al., 2020). Our results revealed that the expression of *GmFLS* (Glyma.18G026500) was rapidly induced in both SS and ST under salt stress, with higher fold changes being observed in ST than in SS. This was consistent with kaempferol content, which was significantly higher in ST than in SS after 12 and 24 h of salt stress treatment (**Figure 9**; **Supplementary Table 8**), suggesting that GmFLS promotes kaempferol synthesis in soybean under salt stress. The CYP81 subfamily comprises several members, however, only a few of them have been identified in plants. For example, the functions of CYP81E in *Medicago truncatula*, SiCYP81Q1 in *Sesamum indicum*, and CYP81Fs in *Arabidopsis thaliana* have been elucidated in previous studies (Liu et al., 2003; Ono et al., 2006; Bednarek et al., 2009; Pfalz et al., 2011). In this study, seven *GmCYP81E* genes were identified in soybean and salt stress significantly induced their expression, which is consistent with the findings in wheat and *Arabidopsis thaliana* (Baruah et al., 2009; Wang et al., 2019). The expression levels of *GmCYP81E* genes in ST were higher than those in SS (**Figure 9**; **Supplementary Table 8**) and correlation analysis revealed that two *GmCYP81E* genes (Glyma.15G156100 and Glyma.11G05180) were strongly correlated with most of the flavonoids identified in this study (**Figure 10A**). In soybean, 2-hydroxy-2,3-dihydrogenistein, which is associated with the flavonoid biosynthetic pathway, can be transformed into 2’-hydroxygenistein by GmHID and GmCYP81E (**Figure 9**). Our study revealed that the expression levels of *GmHID* and *GmCYP81E* genes, which were higher in ST than in SS, were consistent with 2’-hydroxygenistein content (**Figure 9**; **Supplementary Tables 8**, **9**). These results suggest that *GmCYP81E* genes may also have a crucial role in soybean salt stress.

According to previous studies, flavonoid biosynthesis is primarily regulated by the MBW complex that consists of MYB, bHLH, and WD40 transcription factors (Cavallini et al., 2015; Yan et al., 2015; Albert et al., 2018). In this study, a total of 43 TFs were identified based on their strong correlation with flavonoid compounds (**Figure 8**; **Supplementary Table 7**). In soybean, GmMYB176 activates the expression of *GmCHS8*, which promotes isoflavonoid biosynthesis (Yi et al., 2010), while *GmMYB100* has a negative regulatory effect on flavonoid biosynthesis (Yan et al., 2015). In this research, the expression level of *GmMYB100* was downregulated in both ST and SS. Notably, the decrease in *GmMYB100* expression was more pronounced in ST compared to SS at all four time points (**Supplementary Table 5**). Additionally, *GmMYB173* regulates the expression of *GmCHS5*, in turn, leading to increased flavonoid accumulation and enhanced soybean salt tolerance (Pi et al., 2018). The role of MYB transcription factors in regulating flavonoid biosynthesis in other plant species has been documented in other species as well (Liu et al., 2015). In our study, 11 *GmMYBs* were identified by WGCNA with two TFs (Glyma.19G264200 and Glyma.09G032100) showing homologous to *AtMYB78* genes and one TF (Glyma.18G273300) showing homologous to *AtMYB112* genes, and their expression levels in ST were higher than those in SS lines (**Figure 8**; **Supplementary Table 7**). Notably, proteins in subgroup 20, including AtMYB2, AtMYB108, AtMYB112, and AtMYB116, have been identified as regulators of salt stress responses (Kelemen et al., 2015). Furthermore, AtMY112 is associated with flavonoid biosynthesis in *A. thaliana* under salinity stress (Lotkowska et al., 2015). Consequently, *GmMYBs* (Glyma.19G264200, Glyma.09G032100, and Glyma.18G273300) are presumed to be involved in flavonoid synthesis in soybean under salt stress. Moreover, WGCNA revealed three TF genes in the blue module: one *bHLH* (Glyma.08G061300), one *ERF* (Glyma.20G168500), and one *NAC* (Glyma.09G235700). The three genes exhibited higher expression levels in ST than in SS (**Figure 8**; **Supplementary Table 7**). Previous studies have demonstrated that *bHLHs*, *ERFs*, and *NACs* regulate flavonoid biosynthesis in various plant species (Zhou et al., 2015; Sun et al., 2019). Overall, these six TF genes were considered to be major regulators of flavonoid biosynthesis in soybean under salt stress. The results of qRT-PCR were consistent with those obtained from transcriptomic data (**Supplementary Figure 13**), however, further studies should be conducted to elucidate the specific functions of genes involved in flavonoid biosynthesis.

## 5 Conclusion

This study conducted metabolomic and transcriptomic analyses to investigate the key flavonoids and genes associated with flavonoid biosynthesis in soybean under salt stress. A total of 151 flavonoids were identified, with flavones, isoflavones, and flavonols being the most abundant metabolites. The results indicated that the ST plants exhibited higher total flavonoid and Pro contents, but lower MDA content than the SS plants under salt stress, suggesting that ST plants have a strong ROS-scavenging ability. In addition, eight flavonoid synthesis-related genes and six TF genes were identified by WGCNA. The role of GmCYP81E in flavonoid biosynthesis in soybean under salt stress was highlighted. Key flavonoid biosynthesis genes were also identified by CCA and PCA. The results of qRT-PCR were consistent with those obtained from transcriptomic data, which confirmed the accuracy of the transcriptomic data and reliability of the candidate genes. This study provides insights into the flavonoid composition and regulatory molecular mechanisms underlying flavonoid accumulation in soybean under salt stress.

## Data availability statement

The datasets presented in this study are deposited in the NCBI Sequence Read Archive, accession number (BioProject: PRJNA1092863).

## Author contributions

YW: Data curation, Writing – original draft, Writing – review & editing, Conceptualization, Formal analysis, Investigation, Methodology, Project administration, Resources, Software, Visualization. WLiu: Formal analysis, Software, Writing – review & editing, Data curation, Methodology. WLi: Data curation, Writing – review & editing, Formal analysis, Investigation, Validation. CW: Data curation, Writing – review & editing, Investigation, Software. HD: Methodology, Writing – review & editing. RX: Methodology, Writing – review & editing, Supervision. YZ: Writing – review & editing, Conceptualization, Data curation, Writing – original draft, Formal analysis, Project administration, Resources, Validation. LZ: Data curation, Resources, Writing – original draft, Writing – review & editing, Conceptualization, Formal analysis, Funding acquisition, Investigation, Methodology, Project administration.
