# Supplementary material for: Integrative analysis of metabolome and transcriptome reveals regulatory mechanisms of flavonoid biosynthesis in soybean under salt stress
Source: Front Plant Sci. 2024 Jun 18;15:1415867. doi: 10.3389/fpls.2024.1415867 (PMC11217524; doi:10.3389/fpls.2024.1415867)
Supplement: Supplementary file 1 [file Presentation_1.pptx]

## Slide 1
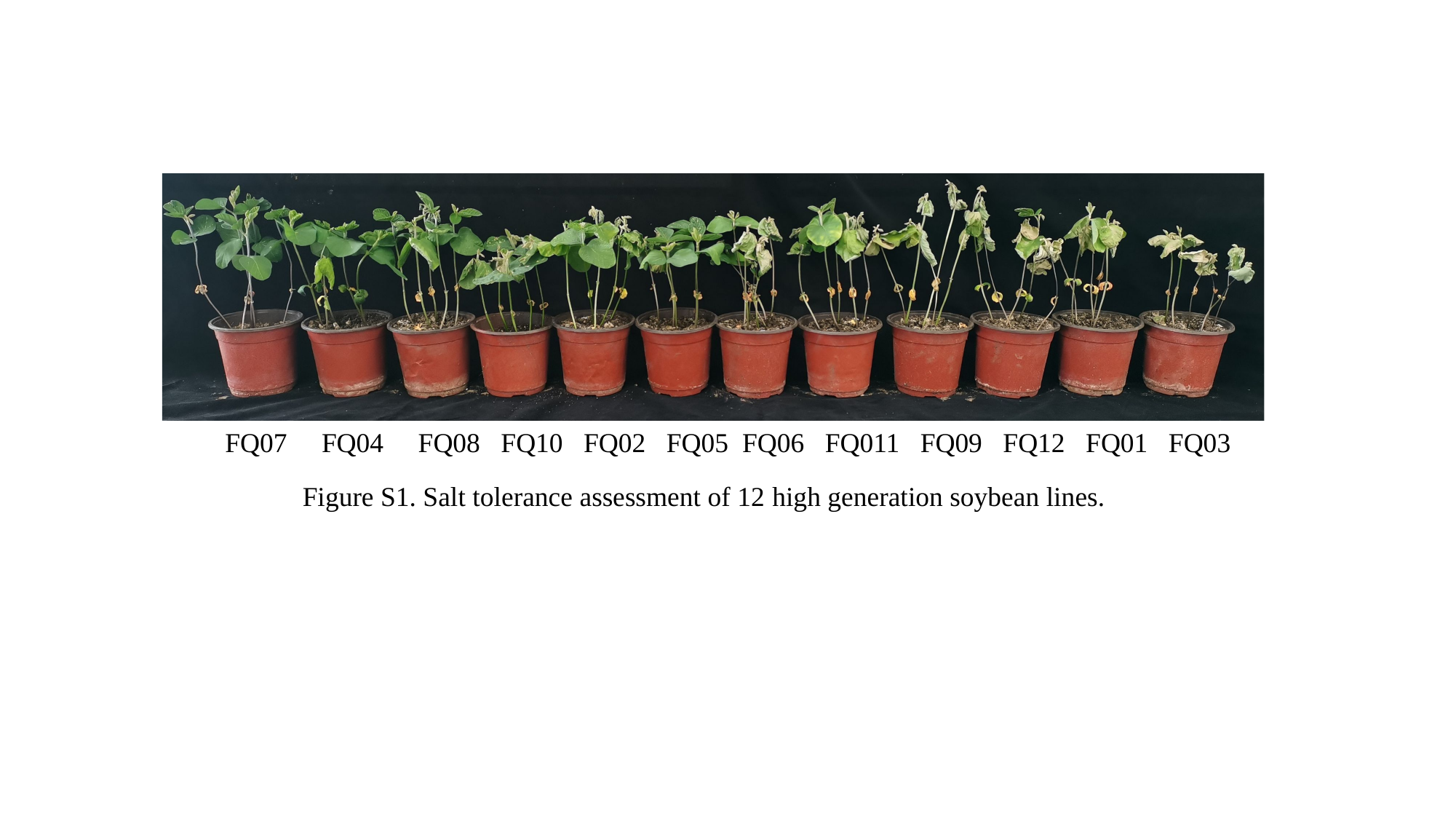

FQ07 FQ04 FQ08 FQ10 FQ02 FQ05 FQ06 FQ011 FQ09 FQ12 FQ01 FQ03
Figure S1. Salt tolerance assessment of 12 high generation soybean lines.

## Slide 2
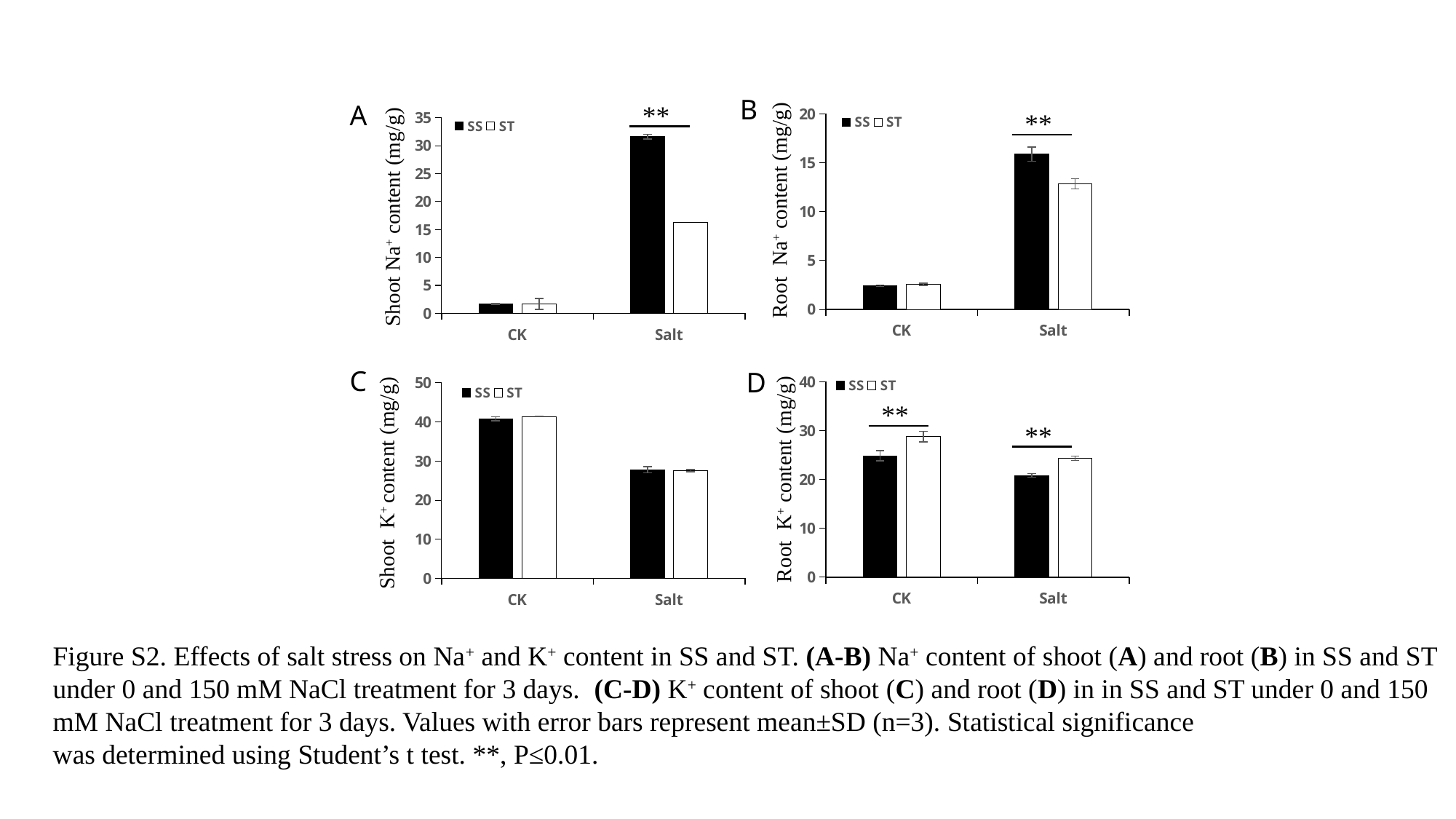

B
**
A
### Chart
| Category | SS | ST |
|---|---|---|
| CK | 2.4116666666666666 | 2.578333333333333 |
| Salt | 15.8725 | 12.849166666666667 |**
### Chart
| Category | SS | ST |
|---|---|---|
| CK | 1.655 | 1.665 |
| Salt | 31.605 | 16.25916666666667 |Root Na+ content (mg/g)
Shoot Na+ content (mg/g)
C
D
### Chart
| Category | SS | ST |
|---|---|---|
| CK | 24.834166666666665 | 28.748333333333335 |
| Salt | 20.824166666666667 | 24.3475 |
### Chart
| Category | SS | ST |
|---|---|---|
| CK | 40.7925 | 41.36416666666667 |
| Salt | 27.771666666666665 | 27.529166666666665 |**
**
Root K+ content (mg/g)
Shoot K+ content (mg/g)
Figure S2. Effects of salt stress on Na+ and K+ content in SS and ST. (A-B) Na+ content of shoot (A) and root (B) in SS and ST under 0 and 150 mM NaCl treatment for 3 days. (C-D) K+ content of shoot (C) and root (D) in in SS and ST under 0 and 150 mM NaCl treatment for 3 days. Values with error bars represent mean±SD (n=3). Statistical significance
was determined using Student’s t test. **, P≤0.01.

## Slide 3
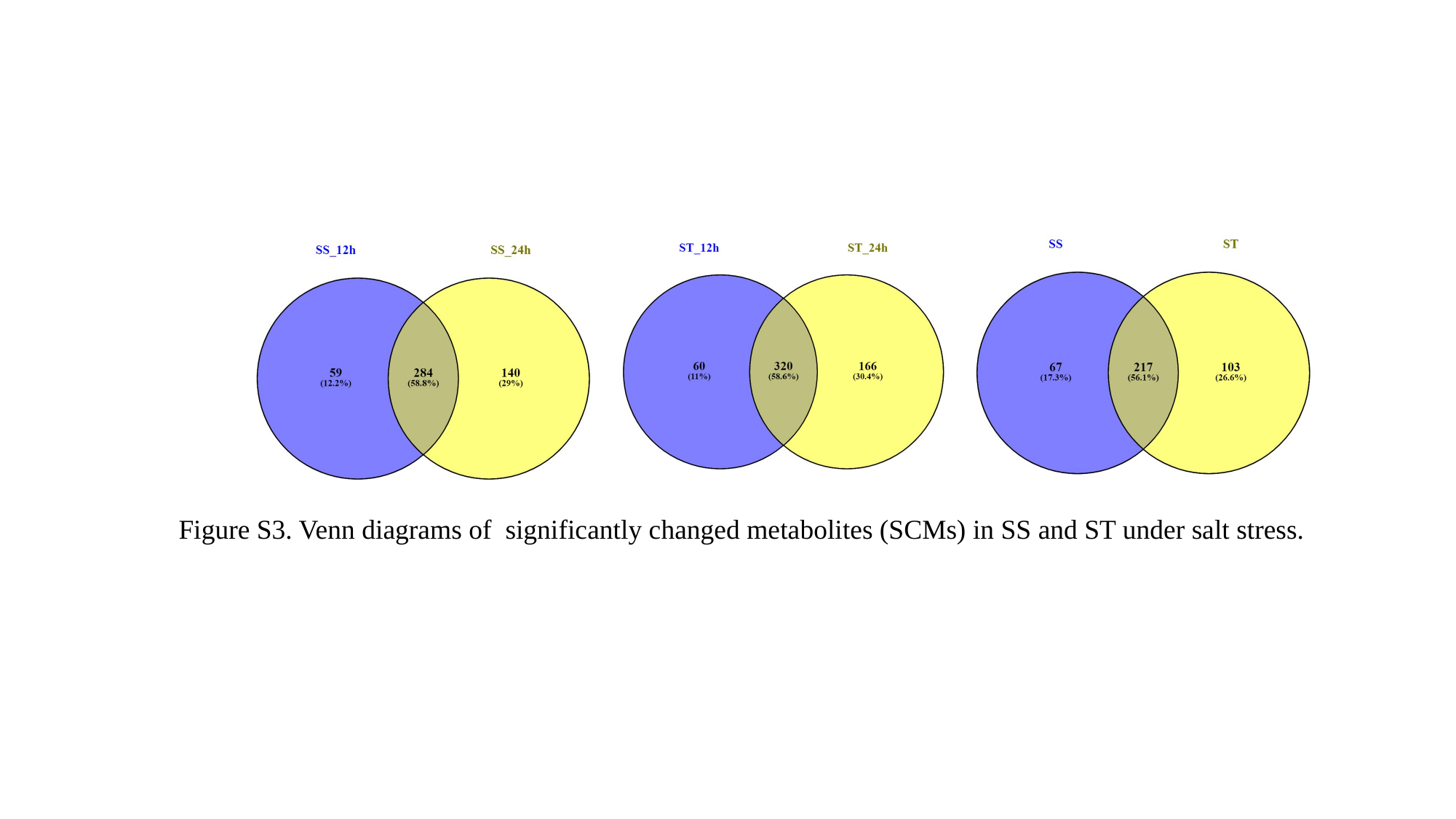

Figure S3. Venn diagrams of significantly changed metabolites (SCMs) in SS and ST under salt stress.

## Slide 4
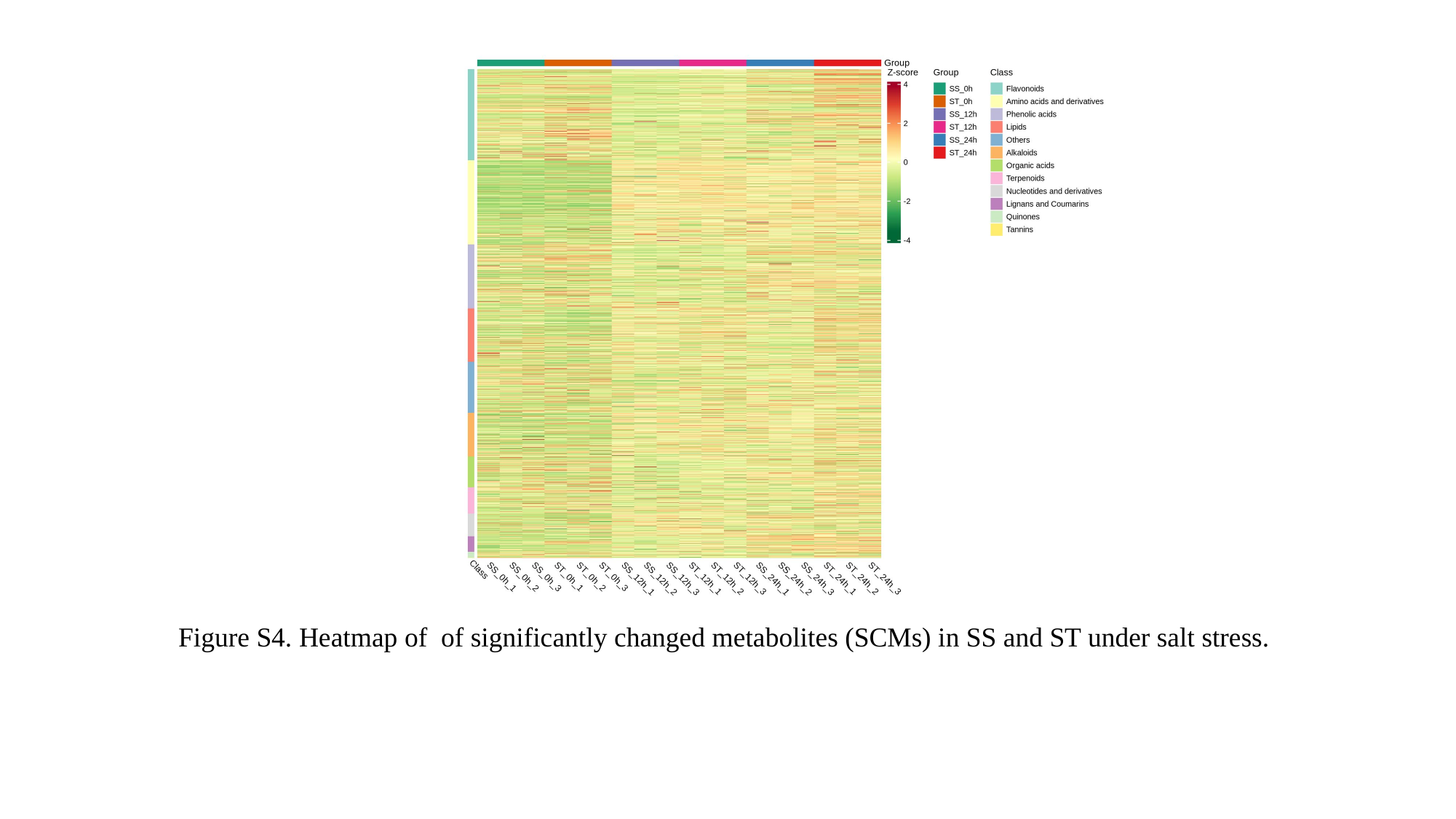

Figure S4. Heatmap of of significantly changed metabolites (SCMs) in SS and ST under salt stress.

## Slide 5
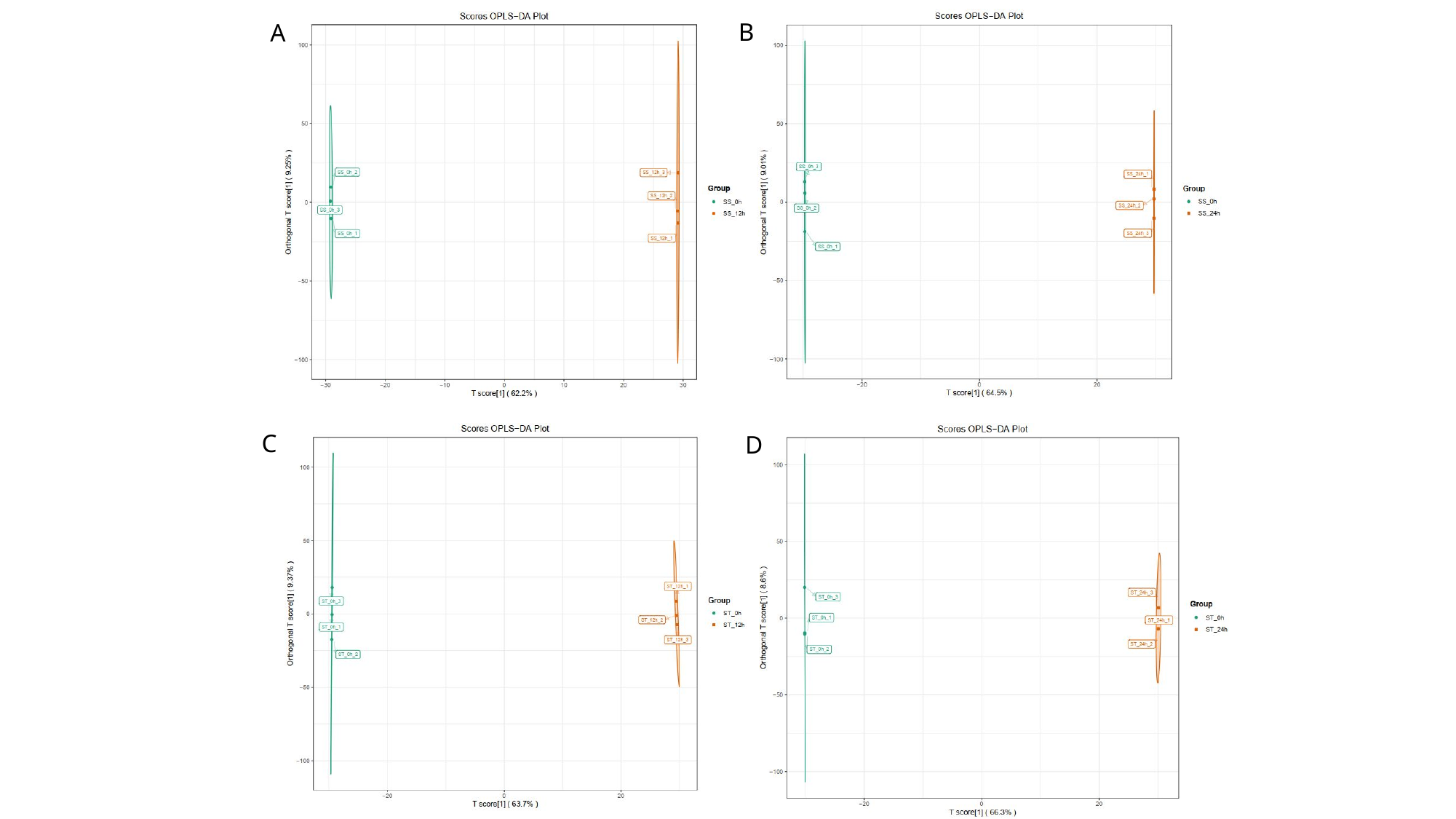

B
A
C
D
Figure S5. OPLS-DA plots of 18 samples in soybean of SS_0 h vs SS_12 h (A), SS_0 h vs. SS_24 h (B), ST_0 h vs ST_12 h (C), ST_0 h vs. ST_24 h (D), respectively.

## Slide 6
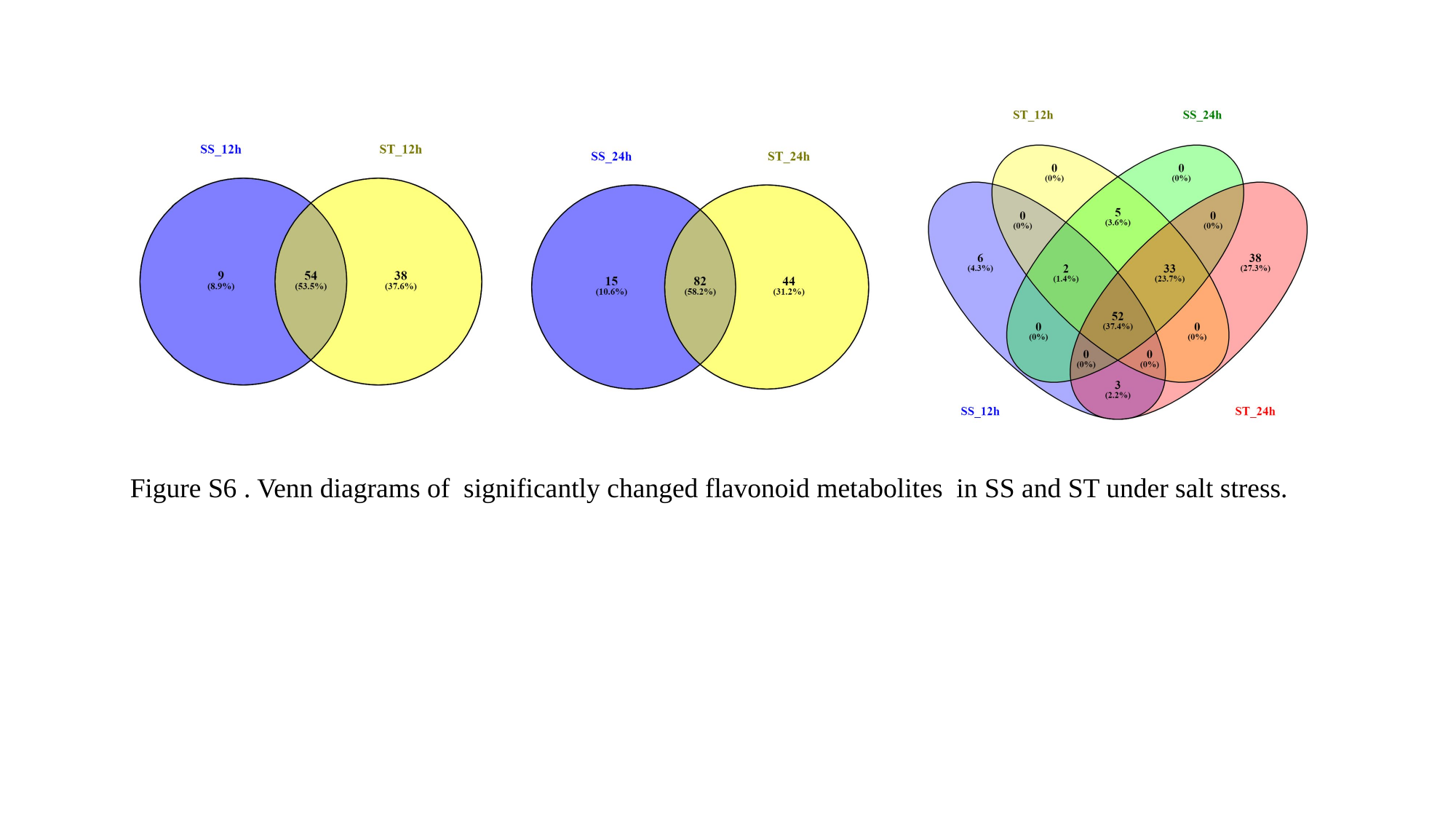

Figure S6 . Venn diagrams of significantly changed flavonoid metabolites in SS and ST under salt stress.

## Slide 7
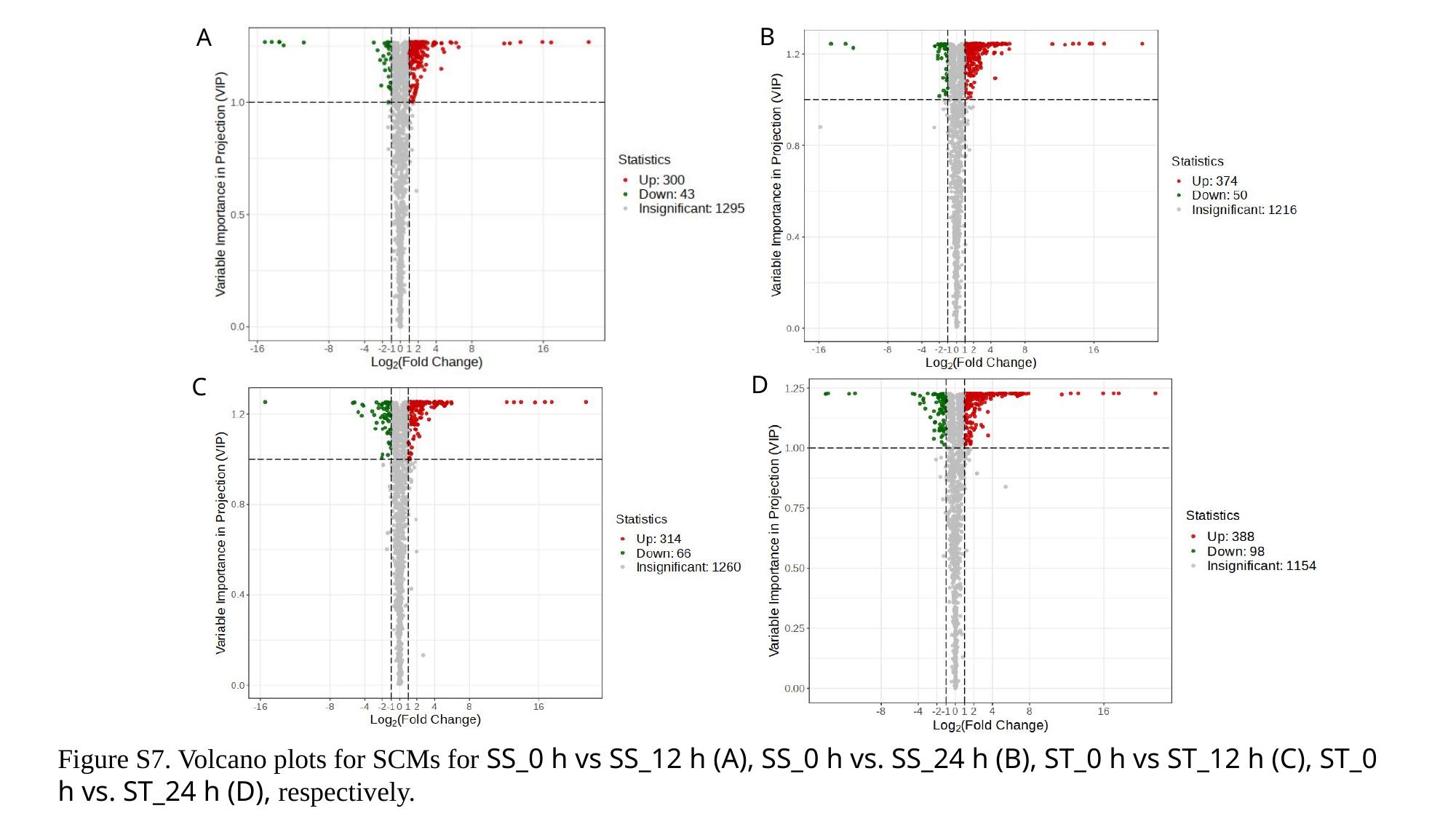

B
A
D
C
Figure S7. Volcano plots for SCMs for SS_0 h vs SS_12 h (A), SS_0 h vs. SS_24 h (B), ST_0 h vs ST_12 h (C), ST_0 h vs. ST_24 h (D), respectively.

## Slide 8
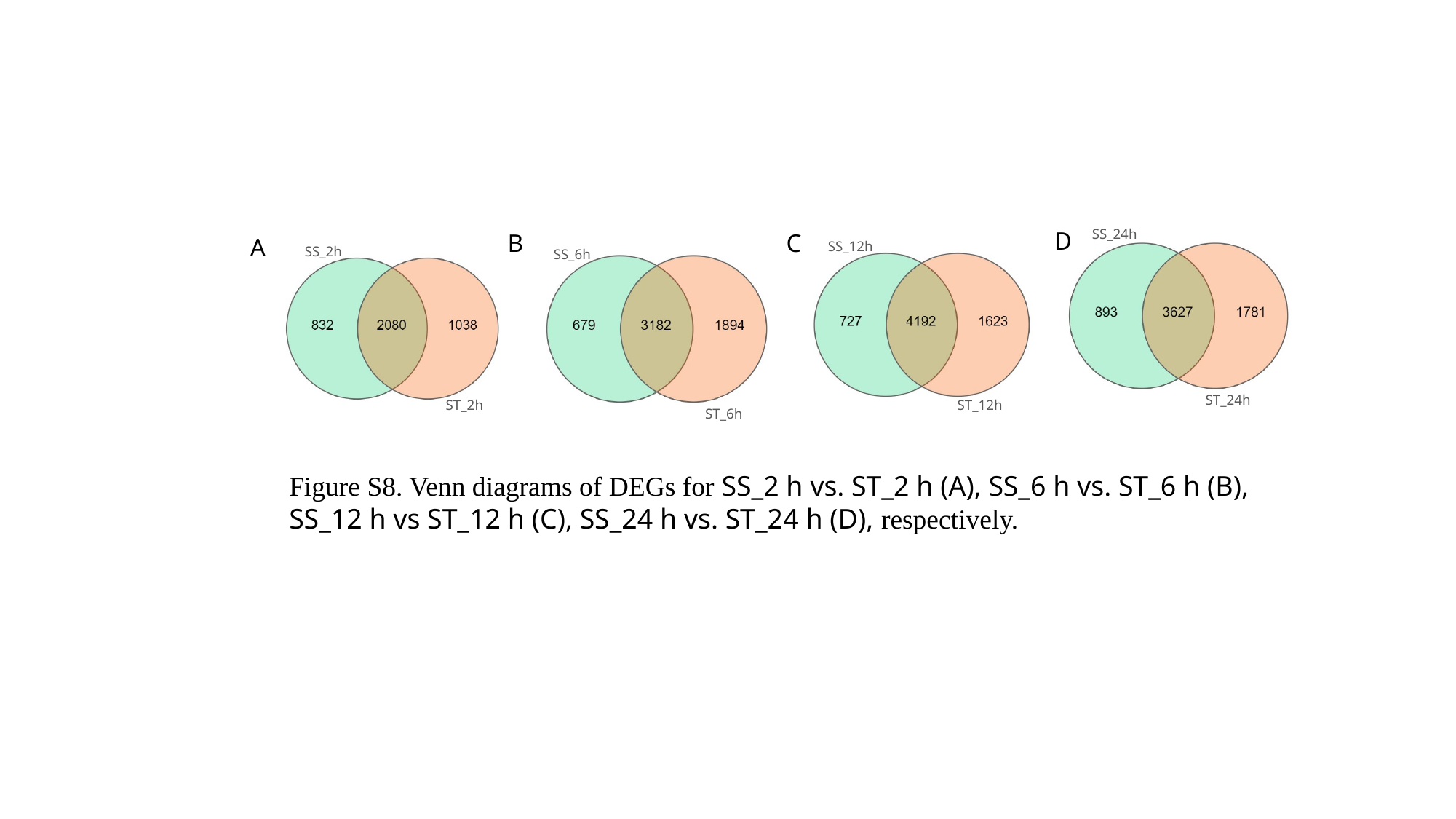

SS_24h
ST_24h
SS_12h
ST_12h
SS_2h
ST_2h
SS_6h
ST_6h
D
B
C
A
Figure S8. Venn diagrams of DEGs for SS_2 h vs. ST_2 h (A), SS_6 h vs. ST_6 h (B), SS_12 h vs ST_12 h (C), SS_24 h vs. ST_24 h (D), respectively.

## Slide 9
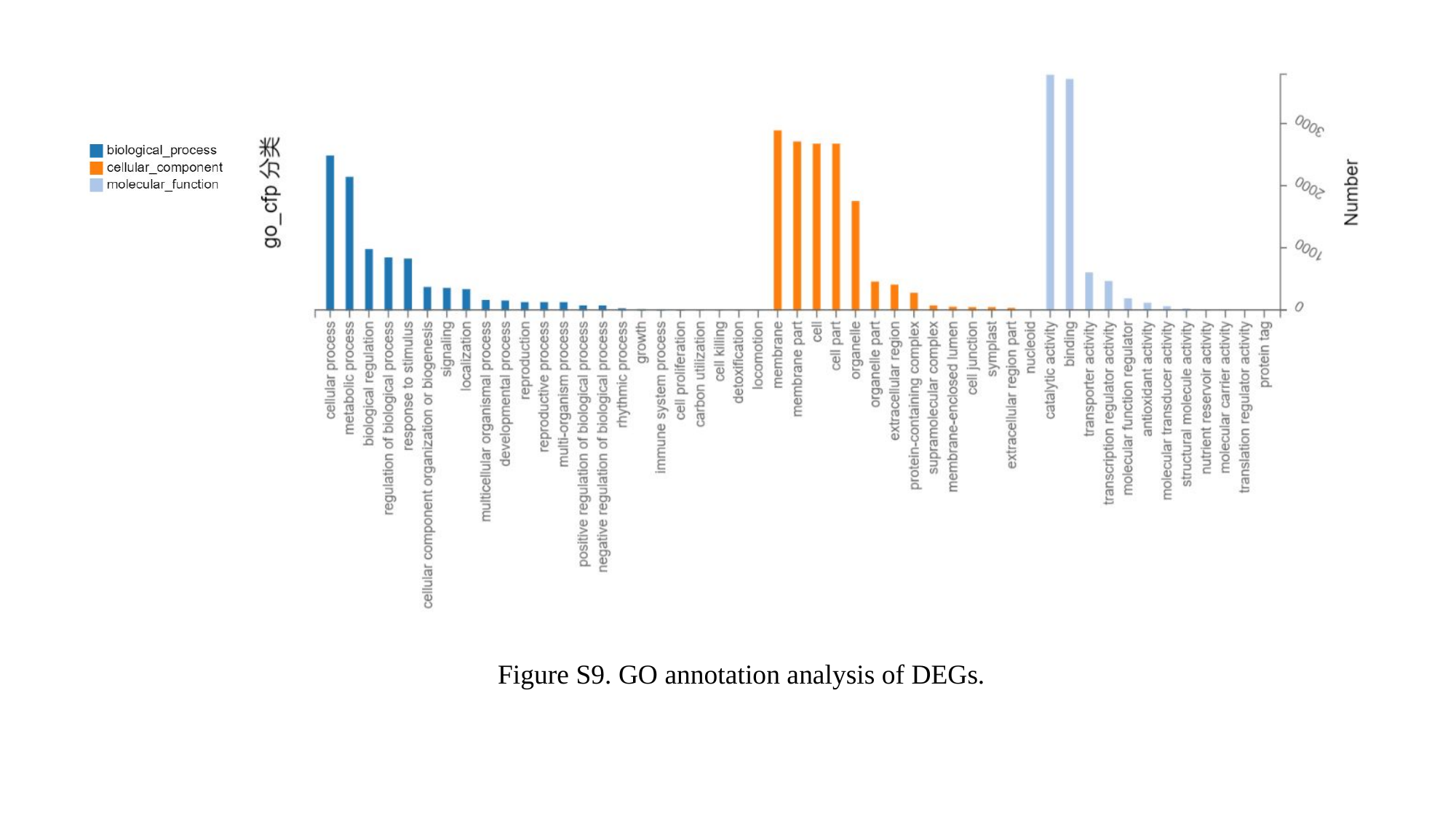

Figure S9. GO annotation analysis of DEGs.

## Slide 10
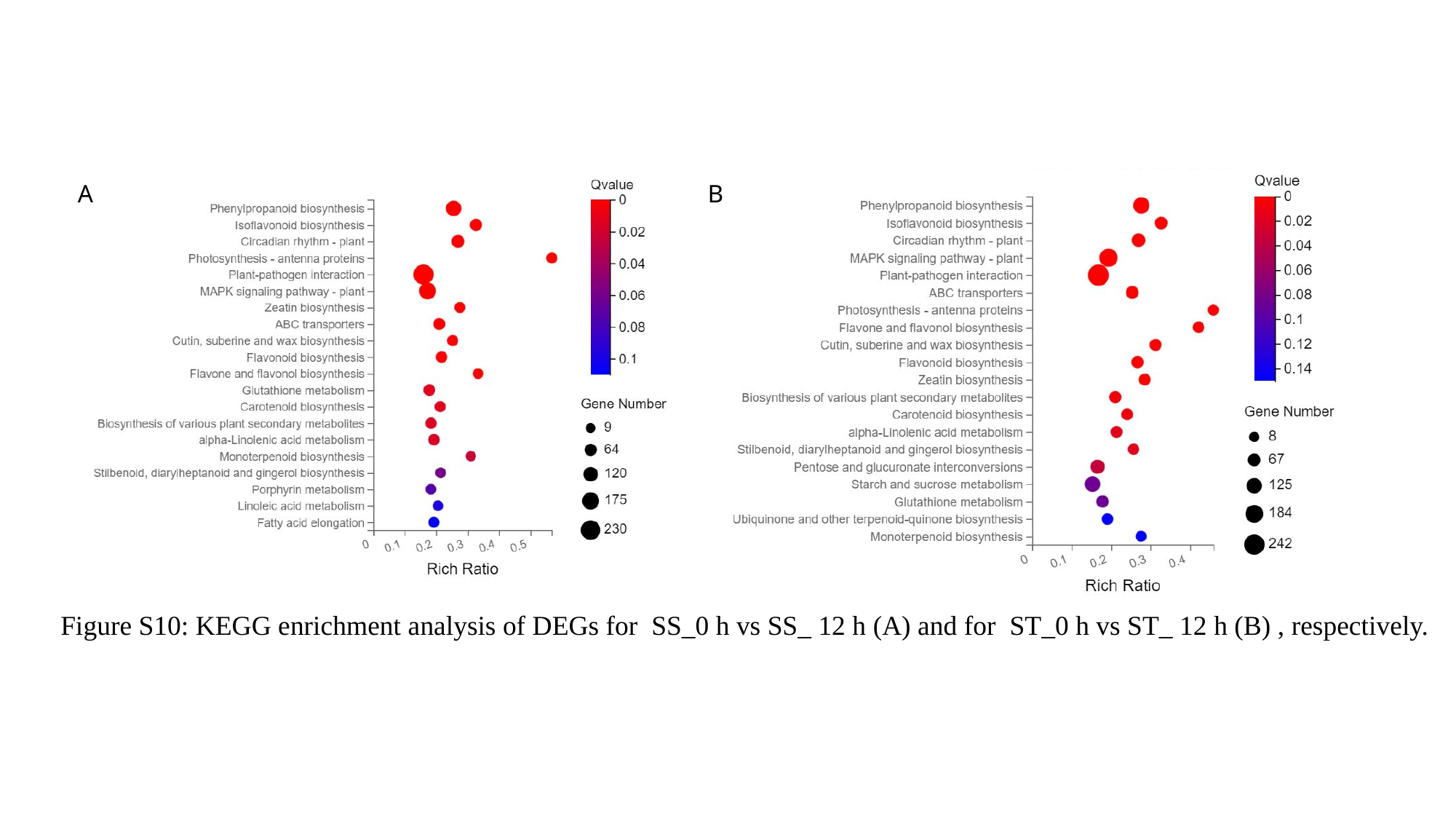

A
B
Figure S10: KEGG enrichment analysis of DEGs for SS_0 h vs SS_ 12 h (A) and for ST_0 h vs ST_ 12 h (B) , respectively.

## Slide 11
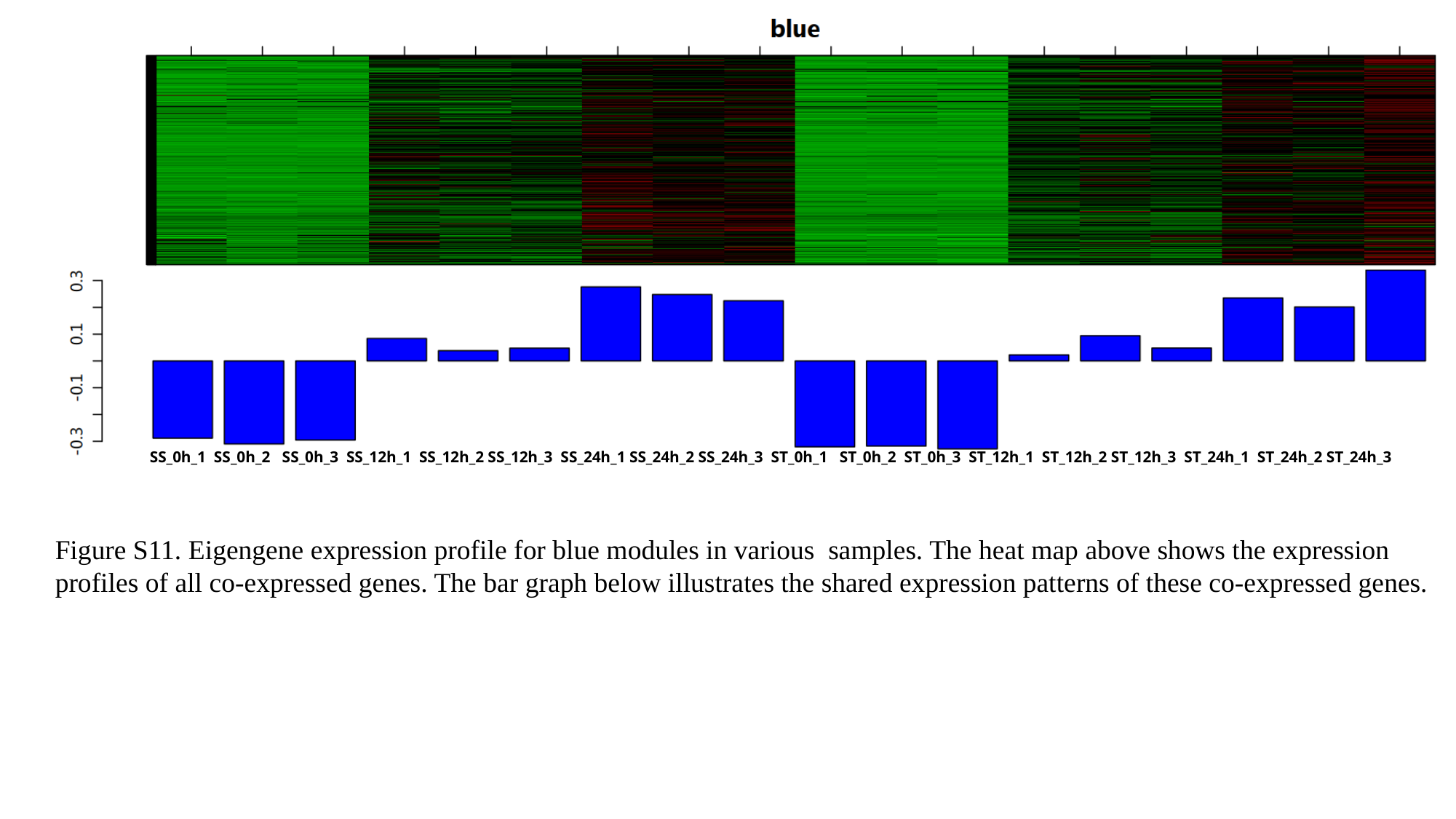

SS_0h_1 SS_0h_2 SS_0h_3 SS_12h_1 SS_12h_2 SS_12h_3 SS_24h_1 SS_24h_2 SS_24h_3 ST_0h_1 ST_0h_2 ST_0h_3 ST_12h_1 ST_12h_2 ST_12h_3 ST_24h_1 ST_24h_2 ST_24h_3
Figure S11. Eigengene expression profile for blue modules in various samples. The heat map above shows the expression profiles of all co-expressed genes. The bar graph below illustrates the shared expression patterns of these co-expressed genes.

## Slide 12
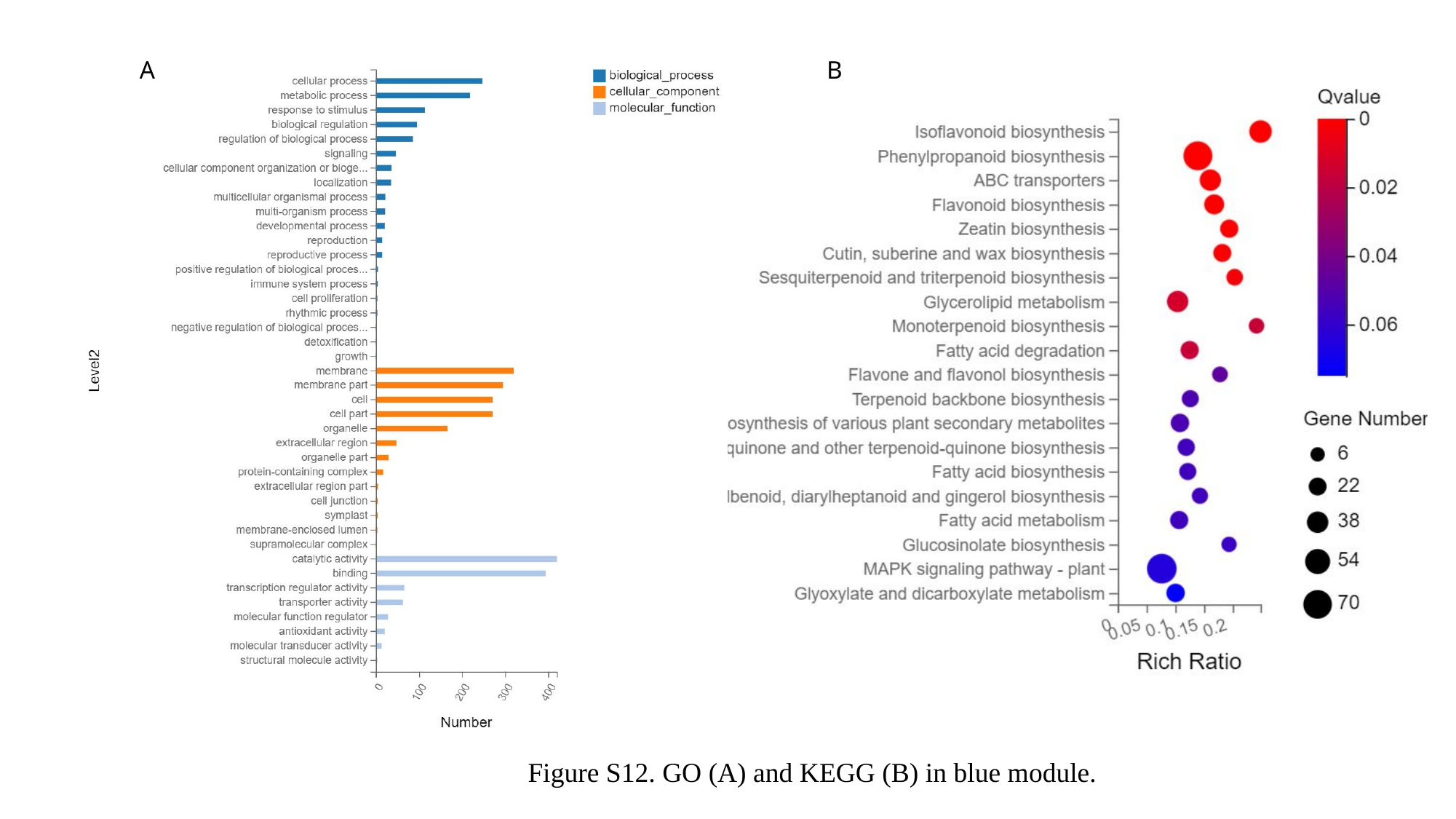

A
B
Figure S12. GO (A) and KEGG (B) in blue module.

## Slide 13
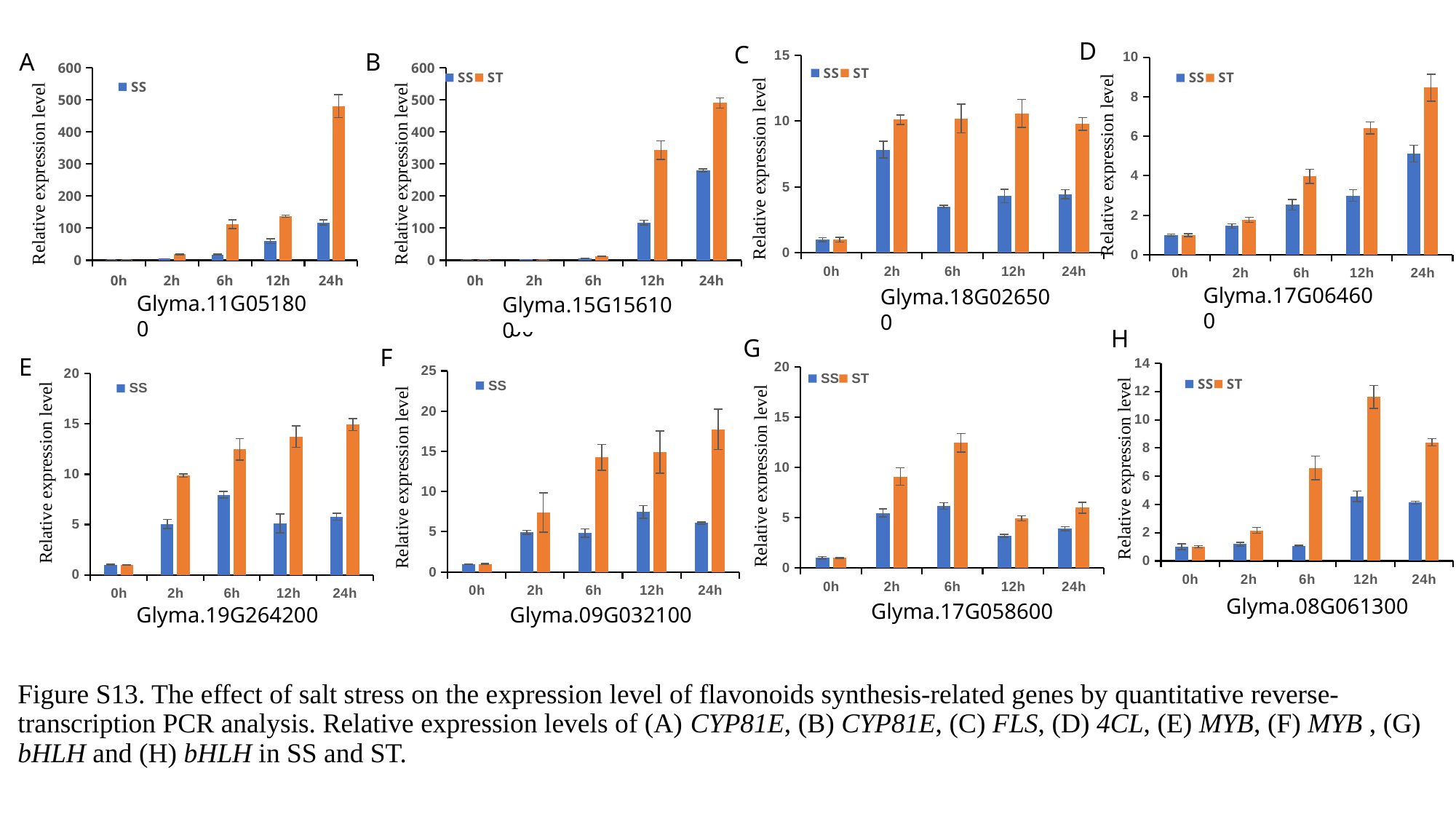

D
C
A
B
### Chart
| Category | SS | ST |
|---|---|---|
| 0h | 1.0081485406583361 | 1.0100147375702233 |
| 2h | 7.834845559461264 | 10.10702227685129 |
| 6h | 3.5153368584833196 | 10.212247153283391 |
| 12h | 4.317497974237553 | 10.589787265616755 |
| 24h | 4.44717500021837 | 9.788799364595784 |
### Chart
| Category | SS | ST |
|---|---|---|
| 0h | 1.0009099601351272 | 1.0018394548768732 |
| 2h | 1.4564329196814345 | 1.7726854059143207 |
| 6h | 2.528761355491067 | 3.9734650695134683 |
| 12h | 2.998689942732566 | 6.4178443213612555 |
| 24h | 5.122465754076116 | 8.455586556421943 |
### Chart
| Category | SS | ST |
|---|---|---|
| 0h | 1.0002075410587326 | 1.0000373278023142 |
| 2h | 4.660359864741603 | 18.341122284476935 |
| 6h | 17.858562152851352 | 112.21760124320629 |
| 12h | 59.57332612118054 | 137.2154751405343 |
| 24h | 117.70620962188652 | 480.8453270772716 |
### Chart
| Category | SS | ST |
|---|---|---|
| 0h | 1.000149808782253 | 1.0003059753072208 |
| 2h | 2.4406706925076826 | 2.2142423892885135 |
| 6h | 5.020507157935762 | 12.411612073500336 |
| 12h | 117.65954704733785 | 343.291733992313 |
| 24h | 280.15866948732497 | 490.1751137406125 | Relative expression level
 Relative expression level
 Relative expression level
 Relative expression level
Glyma.17G064600
Glyma.18G026500
Glyma.11G051800
Glyma.15G156100
Glyma.15G156100
H
G
F
E
### Chart
| Category | SS | ST |
|---|---|---|
| 0h | 1.0154310767177062 | 1.0025081536876272 |
| 2h | 1.1859063271649377 | 2.1599337320565746 |
| 6h | 1.079580109219019 | 6.592339119608874 |
| 12h | 4.573730188197307 | 11.6236338194431 |
| 24h | 4.122829050090577 | 8.400302968062672 |
### Chart
| Category | SS | ST |
|---|---|---|
| 0h | 1.00431092394126 | 1.0004066780819534 |
| 2h | 5.460775784805464 | 9.091932832401282 |
| 6h | 6.1530947057425935 | 12.431690878322103 |
| 12h | 3.1987878793557876 | 4.940230478070898 |
| 24h | 3.892018513158687 | 5.981749313201244 |
### Chart
| Category | SS | ST |
|---|---|---|
| 0h | 1.0001015466641425 | 1.0004852911615247 |
| 2h | 4.929340540259767 | 7.408038432309968 |
| 6h | 4.84046913759485 | 14.25319726367094 |
| 12h | 7.460218621442944 | 14.886480556574716 |
| 24h | 6.091951660612587 | 17.749501796506138 |
### Chart
| Category | SS | ST |
|---|---|---|
| 0h | 1.0096421753184428 | 1.0045182151645184 |
| 2h | 5.055525975135557 | 9.867001094440225 |
| 6h | 7.95399913375967 | 12.47162513380348 |
| 12h | 5.119862352380236 | 13.740021690020631 |
| 24h | 5.782283998521571 | 14.939952120531972 | Relative expression level
 Relative expression level
 Relative expression level
 Relative expression level
Glyma.08G061300
Glyma.17G058600
Glyma.09G032100
Glyma.19G264200
# Figure S13. The effect of salt stress on the expression level of flavonoids synthesis-related genes by quantitative reverse-transcription PCR analysis. Relative expression levels of (A) CYP81E, (B) CYP81E, (C) FLS, (D) 4CL, (E) MYB, (F) MYB , (G) bHLH and (H) bHLH in SS and ST.
